# Photo-Energized MoS_2_/CNT Cathode for High-Performance Li–CO_2_ Batteries in a Wide-Temperature Range

**DOI:** 10.1007/s40820-024-01506-1

**Published:** 2024-09-21

**Authors:** Tingsong Hu, Wenyi Lian, Kang Hu, Qiuju Li, Xueliang Cui, Tengyu Yao, Laifa Shen

**Affiliations:** Jiangsu Key Laboratory of Materials and Technologies for Energy Storage, College of Materials Science and Technology, Nanjing University of Aeronautics and Astronautics, Nanjing, 210016 People’s Republic of China

**Keywords:** Li–CO_2_ batteries, Photo-energized, Wide operation-temperature, Kinetics, MoS_2_

## Abstract

Li–CO_2_ batteries are considered promising energy storage systems in extreme environments such as Mars; however, severe performance degradation will occur at a subzero temperature owning to the sluggish reaction kinetics. Herein, a photo-energized strategy adopting sustainable solar energy in wide working temperature range Li–CO_2_ battery was achieved with a binder-free MoS_2_/carbon nanotube (CNT) photo-electrode as cathode. The unique layered structure and excellent photoelectric properties of MoS_2_ facilitate the abundant generation and rapid transfer of photo-excited carriers, which accelerate the CO_2_ reduction and Li_2_CO_3_ decomposition upon illumination. The illuminated battery at room temperature exhibited high discharge voltage of 2.95 V and mitigated charge voltage of 3.27 V, attaining superior energy efficiency of 90.2% and excellent cycling stability of over 120 cycles. Even at an extremely low temperature of − 30 °C, the battery with same electrolyte can still deliver a small polarization of 0.45 V by the photoelectric and photothermal synergistic mechanism of MoS_2_/CNT cathode. This work demonstrates the promising potential of the photo-energized wide working temperature range Li–CO_2_ battery in addressing the obstacle of charge overpotential and energy efficiency.

**Supplementary Information:**

The online version contains supplementary material available at 10.1007/s40820-024-01506-1.

## Highlights

- The unique layered structure and excellent photoelectric properties of MoS_2_ facilitate the abundant generation and rapid transfer of photo-excited carriers, which accelerate the CO_2_ reduction and Li_2_CO_3_ decomposition upon illumination.
- MoS_2_-based photo-energized Li–CO_2_ battery displays ultra-low charge voltage of 3.27 V, high energy efficiency of 90.2%, superior cycling stability after 120 cycles and high rate capability.
- The low-temperature Li–CO_2_ battery achieves an ultra-low charge voltage of 3.4 V at –30 °C with a round-trip efficiency of 86.6%.

**Supplementary Information:**

The online version contains supplementary material available at 10.1007/s40820-024-01506-1.

## Graphical abstract

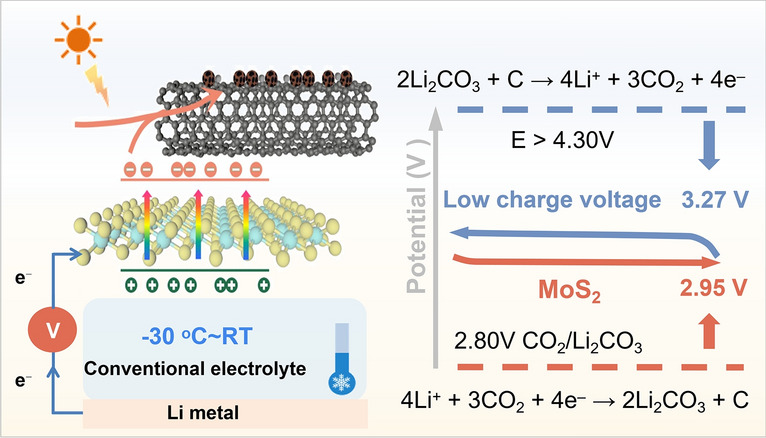

## Introduction

The rechargeable Li–CO_2_ battery emerges as a newly conceptual and promising energy conversion and storage device to alleviate the environmental crisis and energy crisis, which can convert carbon dioxide into sustainable electricity with a standout theoretical specific capacity of 1876 Wh kg^–1^ [1–9]. However, in spite of the above-mentioned favorable factors and promising prospects, the development of Li–CO_2_ battery has been plagued by high voltage gap and slow kinetics of decomposition during charging due to the insulated discharge product Li_2_CO_3_ with high thermodynamic stability [10–12]. In recent years, some advances have been made for Li–CO_2_ batteries with various catalysts including metal, alloy, single atom, and oxide, but their improved voltage gaps were still beyond 1 V and the challenging problem of high overpotential still exists to be addressed [13–17]. In response to this issue, the introduction of energy supplements from the external environment presents a promising strategy for energy conversion and storage [18, 19]. In this way, solar energy, as a clean, abundant and sustainable energy source, has generated wide interest and been adopted to devices for CO_2_ reduction or electricity conversion and storage of electrical energy [20–24].

However, the overall impression from the previous works on electrode design of Li–CO_2_ batteries is confined to operating only at room temperature. For the practical use of Li–CO_2_ batteries in applications, such as mars landing and deep space exploration, low-temperature operation is an essential requirement [25–27]. The decrease of ambient temperature inevitably leads to increased viscosity of electrolyte, increased charge-transfer resistance at the electrode/electrolyte interface, so that more energy is needed to urge the discharge and charge process [28–31]. The electrolyte for low-temperature Li–CO_2_ batteries was replaced by the low-temperature adaptive electrolyte as previous work reported, which limited the application of room temperature [32]. In order to adapt to wide temperature environments, the thermal effect of solar energy could assist Li–CO_2_ batteries without electrolyte replaced in self-heating to meet the requirements [33]. As for photo-energized Li–CO_2_ batteries, photoelectric effect efficiently accelerates the reaction kinetics of electrochemical reduction of CO (COER) by leap of photons-excited electrons, and strong photothermal effect enhances visible light absorption and the conversion of solar energy to heat [34–37]. Therefore, photoelectric and photothermal synergistic mechanism of photo-energized cathode can effectively speed up the interfacial charge transfer of low-temperature environments, but stable cycling at low temperatures remains an urgent issue to be addressed.

In this study, we design a photo-energized binder-free Li–CO_2_ battery with semiconducting 2H–MoS_2_ on carbon nanotube (CNT) conductive substrate (MoS_2_/CNT) as a photocathode to content the requirement of wide temperature range application. Combining DFT calculations and optical properties, tightly integrated MoS_2_/CNT with narrow band gap ensures effective absorption of most visible light and subsequently guarantees abundant generation as well as rapid transfer from MoS_2_ to CNT of photo-excited electrons and holes. Sensitive current response and significantly reduced impedance illustrate the efficient ions diffuse and enhanced reaction kinetics contributing to the excellent electrochemical performance. The Li–CO_2_ battery with MoS_2_/CNT photocathode upon illumination exhibits a higher discharge voltage platform of 2.95 V and the charge voltage down to 3.27 V, leading to high energy efficiency of 90.2% than 74.9% of non-illuminated battery. Benefiting from complete decomposition of insulated discharge products Li_2_CO_3_, the battery shows robust cycle stability over 120 cycles. Due to the graphene-like two-dimensional structure with high specific surface area, MoS_2_ demonstrates excellent photothermal and photoelectric synergistic effects [38, 39]. At an extremely low temperature of − 30 °C, the battery without electrolyte replaced achieves an ultra-low charge voltage of 3.4 V and maintains high energy efficiency of 86.6% by simultaneously promoting the generation of photo-generated charge carriers and heat under illumination.

## Experimental Section

### Chemicals and Materials

CNT paper was purchased from JERNANO, Suzhou. Sodium molybdate dihydrate (Na_2_MoO_4_·2H_2_O) and thiourea (CN_2_H_4_S) were purchased from Macklin. Lithium-air electrolyte (1.0 M LiTFSI in TEGDME) was purchased from DoDoChem, Suzhou. Lithium sheets were purchased from BEIKE, Shenzhen. High-purity CO_2_ gas (99.999%, Nanjing Chuangda Special Gas Factory) was used in all experiment.

### Carbon Nanotube Paper Activation Treatment

The CNT paper used in this experiment was activated before loading MoS_2_ on it. Cut the CNT paper into 2 cm × 4 cm. First, pour 65%–68% concentrated nitric acid into a beaker. The cut CNT paper was completely immersed in concentrated nitric acid, followed by reflux condensation at 90 °C for 9 h. After the acidification treatment, the CNT paper was removed and rinsed several times with deionized water to remove the nitric acid left on the surface of the CNT paper. Finally, the CNT paper was placed on nickel foam in a vacuum drying oven at 60 °C for 12 h.

### MoS_2_/CNT Cathode Preparation

0.121 g Na_2_MoO_4_·2H_2_O and 0.157 g CN_2_H_4_S was dissolved with 20 mL deionized water under stirring for 40 min. Then the solution was transferred to a 30 mL Teflon-lined stainless-steel autoclave. A piece of pre-prepared CNT paper (2 cm × 4 cm) was immersed into the solution and the mixture was sealed and heated in an oven at 200 °C for 24 h. After cooling down to room temperature, the CNT paper was taken out and rinsed with deionized water for several times, followed by being dried in a vacuum oven at 60 °C for 12 h. After heat treatment in a tube furnace at 600 °C for 4 h with a slow ramping rate at 2 °C min^–1^, the MoS_2_/CNT compound film was obtained and cut into 1.13 cm^2^ disks for use as cathode.

### Materials Characterization

The morphologies of samples were characterized by scanning electron microscope (SEM, LYRA3, TESCAN, Czech) equipped with element mapping energy-dispersive spectrometer and TEM (Talos F200X G2, FEI). X-ray diffraction (XRD, X’Pert3 Powder, PAN alytical, Netherlands) was conducted with Cu Kα radiation (*λ* = 0.154178 nm) at a scanning speed of 5° min^–1^ between 10° and 80°. Raman spectra were obtained at an excitation wavelength of 532 nm. Both Raman and PL spectra were collected using long focal length spectrometer (1000 M Series, Horiba, USA). X-ray photoelectron spectroscopy (XPS) measurements were achieved by PHI-5000versaprobe (Thermo Fisher Scientific, USA) with Al Ka (1486.6 eV) as the X-ray source. UV–Vis absorption spectrum was achieved by Lambda 1050 + UV/VIS/NIR Spectrometer (PerkinElmer, USA).

### Photo-Energized Li–CO_2_ Battery Assembly and Electrochemical Measurement

The battery assembly was performed in an Ar-filled glovebox with both O_2_ and H_2_O contents below 0.1 ppm. The Li–CO_2_ battery was assembled in a homemade cell with a gas cavity and a transparent window which guarantees adequate illumination of light. The cell contains Li foil as anode, glass fiber as separator, the obtained MoS_2_/CNT photo-electrode as cathode/separator and a solution of 1.0 M LiTFSI in TEGDME as electrolyte. The MoS_2_/CNT was cut into 1.13 cm^2^ and the effective area for illumination is 0.5 cm^2^. All experiments were performed at 0.05 mL min^–1^ of CO_2_ fixed flow rate. Galvanostatic discharge/charge cycles were conducted on a Land-CT3001A battery-testing system. For these electrochemical tests, the light source for illumination is a 300-W ultraviolet (UV) lamp (MC-PF300C, Merry Change, China), and the illumination intensity on the surface of MoS_2_/CNT electrode is 150 mW cm^–2^. Cyclic voltammetry (CV) curves were measured in a range of 2.0–5.0 V at 5 mV s^–1^. Electrochemical impedance spectroscopy (EIS) data of photo-induced cells were obtained in the range of 100 kHz to 10 mHz. Linear sweep voltammetry (LSV) curves, CV spectra and EIS data were measured via Biologic SP-200 electrochemical workstation. The ionic conductivity (σ) of FGPE was calculated through the equation $\sigma =\frac{d}{RS}$, where *d* (0.06 cm) and *S* (1.54 cm^2^) belong to the thickness and area of diaphragm, respectively. *R* represents the bulk resistance of electrolyte originating from the EIS of SS//SS symmetrical battery.

### Low-Temperature Li–CO_2_ Battery Assembly and Temperature Measurement

The low-temperature Li–CO_2_ battery is assembled in a CR2032 buckle type battery shell with total area of 0.29 cm^2^ holes punched on the cathode shell as gas diffusion and light receiving holes. The battery contains Li foil as anode, glass fiber as separator, the obtained MoS_2_/CNT photo-electrode as cathode/separator and a solution of 1.0 M LiTFSI in TEGDME as electrolyte. The battery is installed into the temperature control device, which includes a temperature detector at the bottom of the battery and an electric heating/liquid nitrogen cooling component. Above the battery is an air chamber and optical window, with a gas path in the device that can continuously introduce CO_2_. The surface temperature distribution of photo-electrode was obtained by taking an infrared (IR) lattice thermal imager. A thermal imager of Guangyun electron GY-MCU90640 model was used for image acquisition.

## Result and Discussion

### Synthesis and Analysis of Binder-Free MoS_2_/CNT Photo-Electrode

**Fig. 1:**
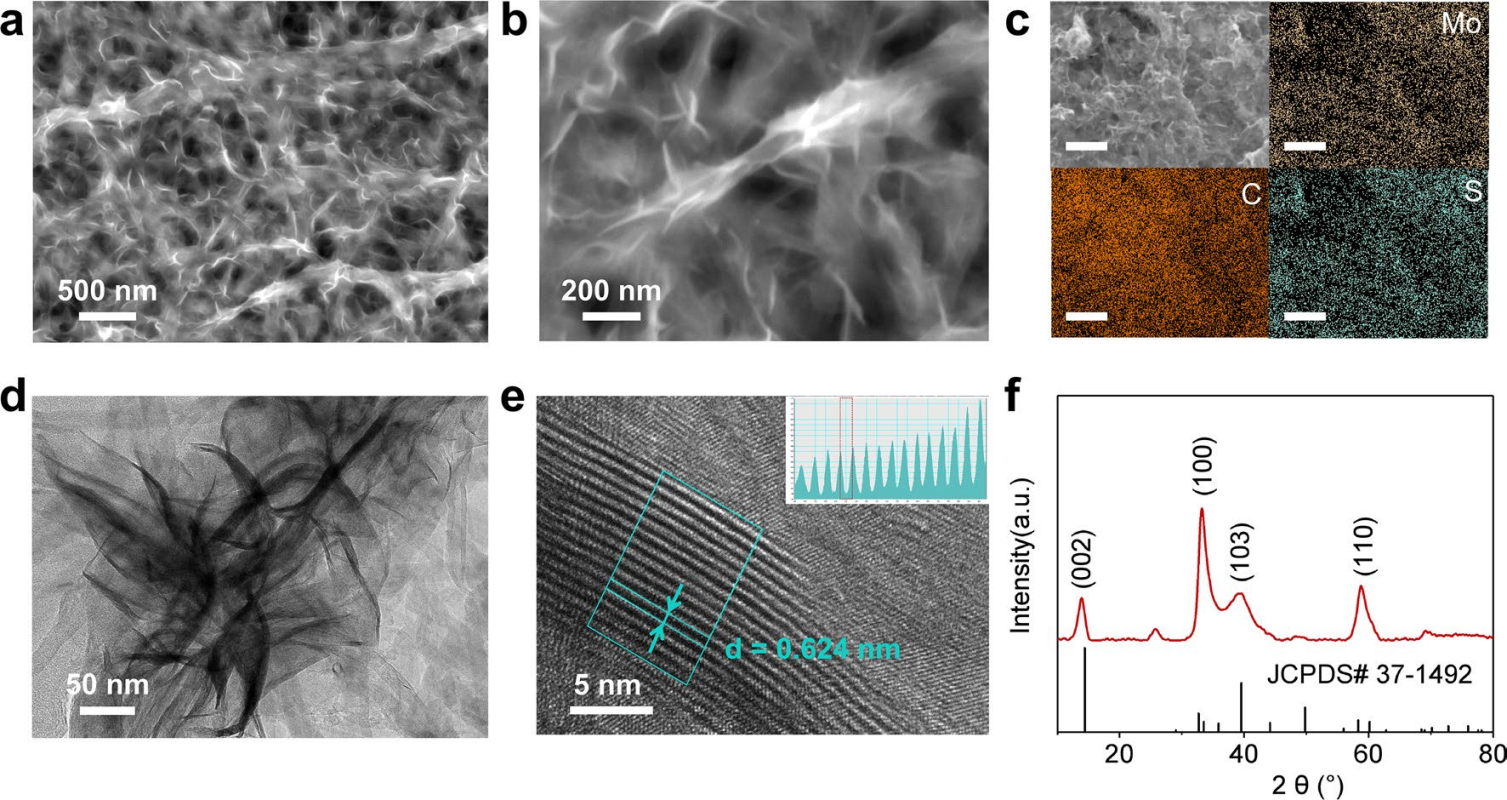
**a**, **b** Scanning electron microscopy images of MoS_2_/CNT. **c** Energy-dispersive spectroscopy images of C, Mo, and S of MoS_2_/CNT. **d**, **e** Transmission electron microscopy images of MoS_2_/CNT. **f** X-ray diffraction patterns of the MoS_2_/CNT cathode

With the objective of highly effective conductivity carriers and maximized separation of the photo-generated electrons/holes, a tightly integrated and binder-free MoS_2_/CNT photo-electrode was designed and prepared according to a hydrothermal synthesis strategy (Fig. S1) [40]. The interior structure of the MoS_2_/CNT was observed via SEM. Compare with the acquired pristine CNT without impurity (Fig. S2), as-synthesized MoS_2_/CNT image (Fig. 1a) exhibits clearly visible MoS_2_ nanosheets growing on carbon fibers. The random-orientated MoS_2_ intercrossing with each other guarantees a porous and gas-permeable nanostructure with abundant reaction sites. The higher-magnification microscopy image in Fig. 1b demonstrates that tubular carbon fibers were tightly wrapped by MoS_2_ nanosheets via hydrothermal and thermal treatment with uniform diameters of around 50–100 nm. The energy-dispersive spectroscopy (EDS) of MoS_2_/CNT reveals that MoS_2_ homogeneously distributed on the CNTs without any observable MoS_2_ nanoclusters (Fig. 1c). Avoiding the accumulation and agglomeration of bulk MoS_2_, two-dimensional structural photocatalysts growing directly onto the conductive substrate enables not only efficient diffusion of CO_2_ but full penetration of electrolyte. Moreover, the adequately contacted heterostructure with high specific surface area provides abundant redox reaction sites and rapid transport of photo-generated carriers. The transmission electron microscopy (TEM) image in Fig. 1d demonstrates that MoS_2_ nanosheets uniformly grew along the intercrossed CNTs, which corresponds the SEM and EDS results. As shown in Fig. 1e, the high-magnification TEM image presents the multilayer structural MoS_2_ nanosheets with a clear lattice spacing of 0.624 nm, which is consistent with the *d*-spacing in the (002) direction of 2H–MoS_2_ [41].

The sample identification and crystallographic structure was characterized by XRD. As shown in Fig. 1f, diffraction peaks locate at 14.0°, 33.2°, 39.3°, and 58.8°, respectively, correspond to the values of (002), (100), (103), and (110) crystal planes of hexagonal MoS_2_ in the standard card (JPCDS #37-1492) [42]. The Raman spectra in Fig. S3 illustrates that the major peaks at 380 and 405 cm^–1^ are consistent with the in-plane and an out-of-plane typical ${E}_{2g}^{1}$ and ${A}_{1g}$ vibrational modes, which confirm the 2H–MoS_2_ phase [43]. The precise chemical states of Mo and S in MoS_2_/CNT were identified by XPS in Fig. S4. The characteristic peaks at 232.78 and 229.68 eV are assigned to the emission from the electrons of Mo 3*d*_3/2_ and Mo 3*d*_5/2_, respectively. In addition to a pair of characteristic peaks observed at 232.78 and 229.68 eV belonging to Mo^4+^, a small peak located at 236.08 eV would be labeled as Mo^6+^ 3*d*_3/2_, and the appearance of Mo^6+^ indicates the surface oxidation of molybdenum trioxide [44]. The peak detected at 226.78 eV is classified as the S–S bond from the residual sulfur that has not reacted with molybdenum. In the S–2*p* spectrum, the peaks observed at 163.68 and 162.48 eV are assigned to S 2*p*_1/2_ and S 2*p*_3/2_, respectively, confirming the above result of the existence and elemental state of MoS_2_ [45].

**Fig. 2:**
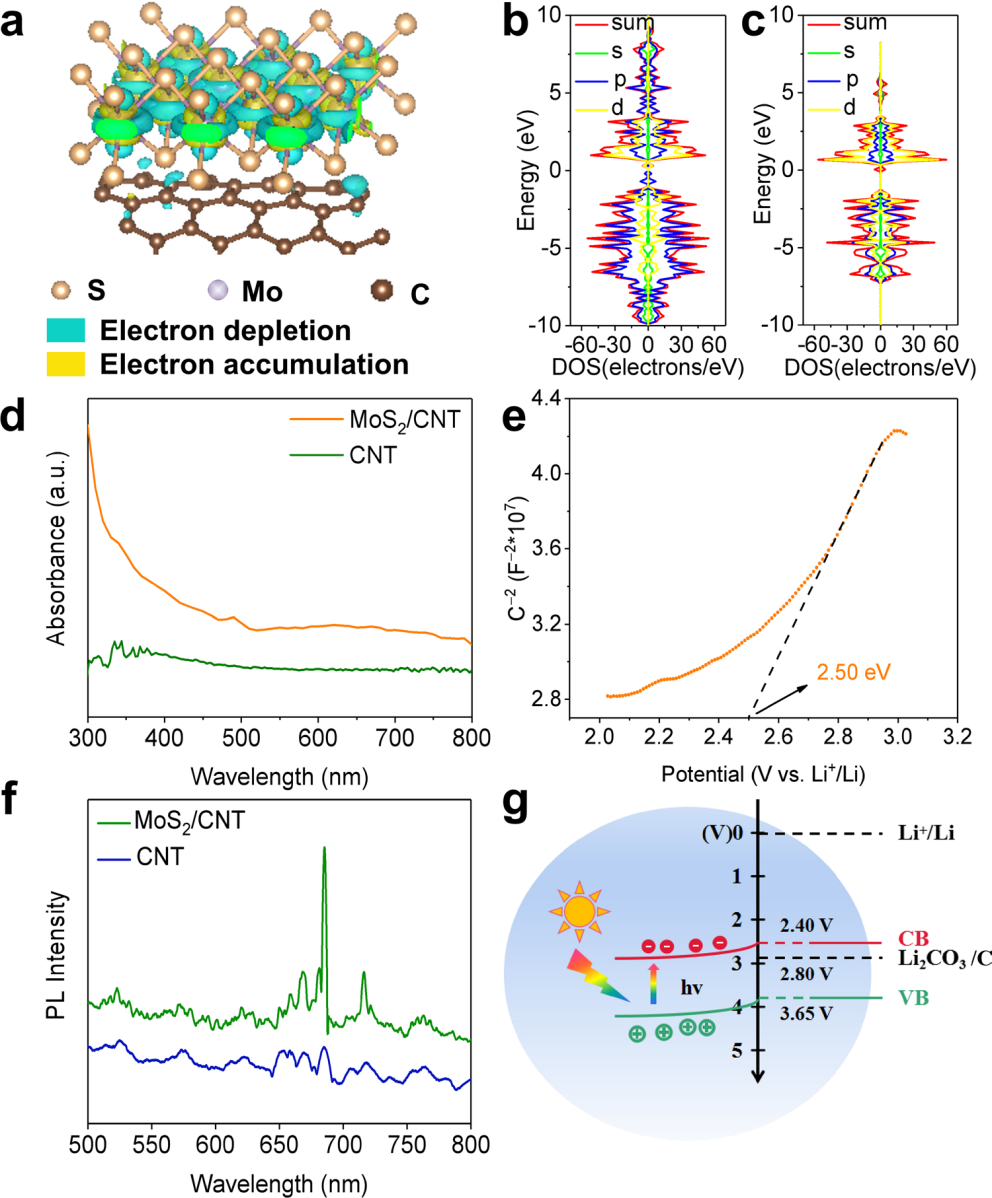
**a** Charge density plot of MoS_2_/CNT. Density of States plot of **b** MoS_2_ and **c** MoS_2_/CNT. **d** UV–Vis absorption spectra of MoS_2_/CNT and CNT. **e** Mott-Schottky spectra of MoS_2_/CNT. **f** Photoluminescence spectroscopy spectra of CNT and MoS_2_/CNT. **g** Working mechanism and energy levels of the photo-energized Li–CO_2_ battery based on the MoS_2_/CNT cathode

Density functional theory (DFT) calculations were carried out to get insight into the electronic behaviors of MoS_2_/CNT. The electron density of states was performed to show the electron-rich regions at the S units of MoS_2_ and the electron-depletion regions at the CNT part, implying the electron spontaneous redistribution from MoS_2_ to CNT (Fig. 2a) [46, 47]. Moreover, the density of states (DOS) and partial density of states (PDOS) of was calculated to analyze the interfacial electronic structures of MoS_2_ [48]. The DOS results further demonstrate equal amount of spin-up and spin-down electrons, illustrating the structural stability of electrons in pristine MoS_2_ and MoS_2_/CNT. Different from the DOS of pristine MoS_2_ structure, two new peaks from 0.85 to 0.02 eV in p orbit appear near Fermi level, implying that the introduction of CNT boost electronic migration via enabling more available electron states near Fermi level for MoS_2_ (Fig. 2b, c). The PDOS of pristine single-layer MoS_2_ and MoS_2_/CNT composite in Fig. S5 illustrates that the valance band maximum is contributed by the d orbit of Mo and p orbit of S, while conductive band minimum is dominated by S–3*p* state. In this state, Fermi energy level located in the interval of zero value without passing-through electron state and the electron state near Fermi level is primarily composed of Mo–4d, indicating the semiconducting properties of pristine MoS_2_ and MoS_2_/CNT. Both pristine MoS_2_ and MoS_2_ combining with CNT are primarily contributed by Mo–4*d* state and S–3*p* state. From 7 to 1 eV of the VB, 4*d* orbit of Mo overlap with 3*p* orbit of S, implying the presence of orbital hybridization in MoS_2_ [49].

To investigate the effect of light on photo-electrode, the optical properties including light-harvesting ability, band structure and photo-generated carriers separation efficiency were further analyzed. Figure 2d shows the UV–Vis absorption in the range of 300–800 nm, in which MoS_2_/CNT demonstrates stronger absorption intensity and distinct absorption peak compared with CNT. In order to evaluate the band gap, Tauc plot (Fig. S6) corresponding to the UV–Vis absorption spectrum was carried out, thus deriving an optical energy gap of 1.25 eV, which is consistent with above results of multilayer 2H–MoS_2_. As shown in Fig. 2e, the positive slope in the Mott–Schottky (M–S) plot illustrates the n-type semiconducting nature of the MoS_2_/CNT cathode and an estimated flat band potential of 2.50 V versus Li^+^/Li which is more positive by about 0.1 V than the CB. Combining the valves of band gap and CB, the VB edges of MoS_2_/CNT is calculated to be 3.74 V versus Li^+^/Li. As for the generation of photo-generated carriers, the photoluminescence spectroscopy (PL) images show that compared with CNT, MoS_2_/CNT was observed with obvious PL peaks, indicating that the presence of MoS_2_ generate electron/hole pairs on cathode under illumination (Fig. 2f). The fluorescence lifetime spectra results illustrate that the interaction of MoS_2_/CNT prolongs the fluorescence lifetime of photo-generated carriers from MoS_2_, which provides evidence for more efficient electron transfer attributed to the excellent electrical conductivity of CNT (Fig. S7). As shown in Fig. 2g, the MoS_2_/CNT photo-electrode satisfies the basic conditions for light-promoted Li–CO_2_ batteries: the potential of the evolution of CO_2_ and Li_2_CO_3_/C (2.80 V vs Li^+^/Li) lies between the CB and VB potentials of photocathode. We conducted first principles calculations using DFT to investigate the dynamic processes under both light and non-light conditions (Tables S1, S2 and Fig. S8). Reaction a1 is the rate-determining step during the charging process, and the Gibbs free energy of this reaction is 4.6250 eV in the absence of light. After applying light, the energy barrier decreases by 0.2965 eV. During the discharge process, reaction b5 is the rate-determining step, and the Gibbs free energy under non-light conditions is 6.4143 eV. After applying light, the energy barrier decreased by 0.1324 eV. The above results present that the effective generation, separation and transfer of photo-generated electrons/holes on MoS_2_/CNT cathode, implying promising photo-energized electrochemical performance during the reaction in Li–CO_2_ battery for energy conversion and storage [50, 51].

### Electrochemical Properties of MoS_2_/CNT Photocathode

**Fig. 3:**
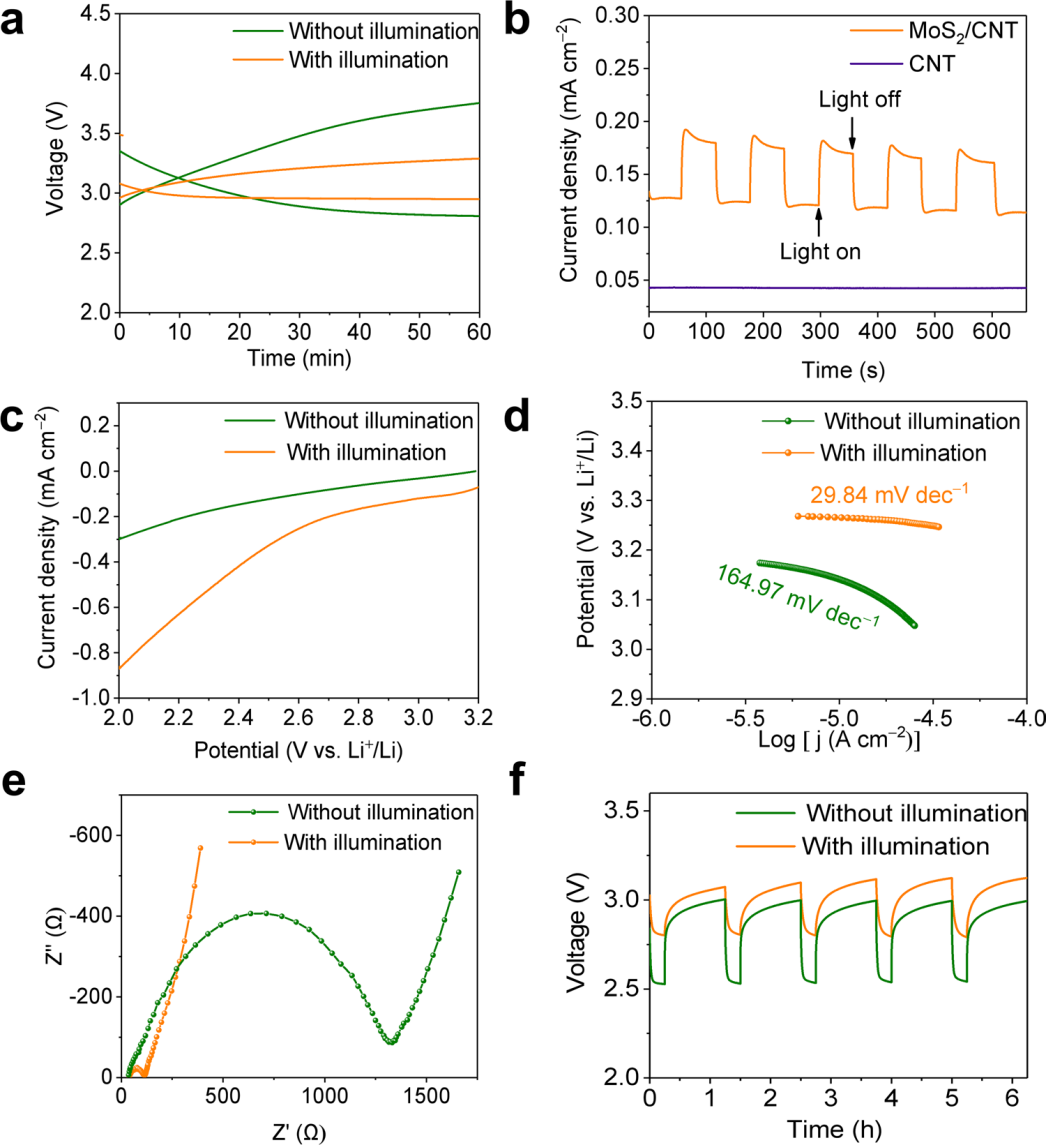
**a** First discharge and charge curves of the Li–CO_2_ battery based on the MoS_2_/CNT cathode with and without illumination at 0.01 mA cm^–2^. **b** Photocurrent response of of MoS_2_ and MoS_2_/CNT. **c** Linear sweep voltammetry curves in CO_2_ reduction process at 5 mV s^–1^ and **d** corresponding Tafel curves, **e** electrochemical impedance spectroscopy spectra, **f** Galvanostatic intermittent titration spectra during discharge of Li–CO_2_ battery with MoS_2_/CNT cathode in the presence and absence of illumination

The photo-energized Li–CO_2_ battery was assembled with a MoS_2_/CNT photocathode, a lithium anode. A Xe lamp with a power density of about 100 mW cm^–2^ was utilized as light source, providing photoenergy with a wavelength range from 380 to 780 nm. CV curves of Li–CO_2_ batteries with MoS_2_/CNT cathode in Ar or CO_2_ depicts significantly larger area capacity in CO_2_ atmosphere than that in Ar (Fig. S9), revealing the conversion and reaction of CO_2_ inside the cell. Figure 3a shows the discharge and charge plots of the MoS_2_/CNT-based Li–CO_2_ battery with and without illumination. As expected, the Li–CO_2_ battery with MoS_2_/CNT photocathode under illumination condition exhibits a higher discharge voltage platform of 2.95 V at 0.01 mA cm^–2^ than that in the dark (2.81 V) which is extremely close to the theoretical discharge voltage of 2.80 V. During reverse charging process, the charge voltage with the introduction of light is down to 3.27 V, which is 0.48 V lower than that without illumination (3.75 V), leading to high energy efficiency of 90.2% than 74.9% of non-illuminated battery. These results indicate that the photo-generated carriers accelerate the reaction in the Li–CO_2_ battery with the conversion of photoenergy. The photoelectric response properties of the cathode were evaluated by photocurrent response. Compared with bare CNT, significant photocurrent responses of MoS_2_/CNT were observed upon on–off cycling irradiation from illumination (Fig. 3b). The illuminated current density of photocathode goes rapidly up to the platform of the maximum value and maintain basically steady, illustrating that the presence of MoS_2_ enables the conversion from light energy to electrical energy in Li–CO_2_ battery.

In order to further investigate the effect of illumination, LSV of Li–CO_2_ batteries was evaluated. Under illumination, MoS_2_/CNT exhibits a more pronounced current density than that under no illumination in reduction process, indicating its enhanced dynamic kinetics and conductivity during discharge/charge process (Figs. 3c and S10a). The Tafel slope inferred from the LSV data demonstrates that the photo-assisted value of 29.84 mV dec^–1^ is much smaller than that under no illumination (164.97 mV dec^–1^) (Fig. 3d). For the opposite oxidation process, a similar result is observed that the slope with illumination is smaller than that in the dark, confirming better oxidation kinetics ascribed to the contribution of photo-generated carriers (Fig. S9b).

The EIS was implemented to further evaluate ion transport properties of the Li–CO_2_ battery with the effect of illumination. Consistently, the plot of EIS in Fig. 3e shows a much smaller impedance of illuminated MoS_2_/CNT cathode than that under no illumination, which illustrates the rapid ions diffuse in battery. Galvanostatic intermittent titration tests (GITT) during discharge and charge was performed to further explore the positive effect of solar energy on the catalytic performance of the photocathode. During the discharge process, the overpotential (0.25 V) of the photo-energized battery is significantly lower than that of around 0.47 V upon the dark condition, implying the compensation of the internal generated photovoltage for the high overpotential in the light-treated Li–CO_2_ battery (Fig. 3f). The reverse charging process also exhibits consistent lower overpotential, revealing that abundant photo-generated electrons from photocathode facilitate the evolution of insulating discharge products (Fig. S11). Above results suggest that solar energy is converted into electrical energy storage during discharge and compensates for the high potential required for product decomposition, which promotes the intrinsic kinetics of Li–CO_2_ battery.

### Reversibility of CORR/COER and Analysis of the Discharge Products

**Fig. 4:**
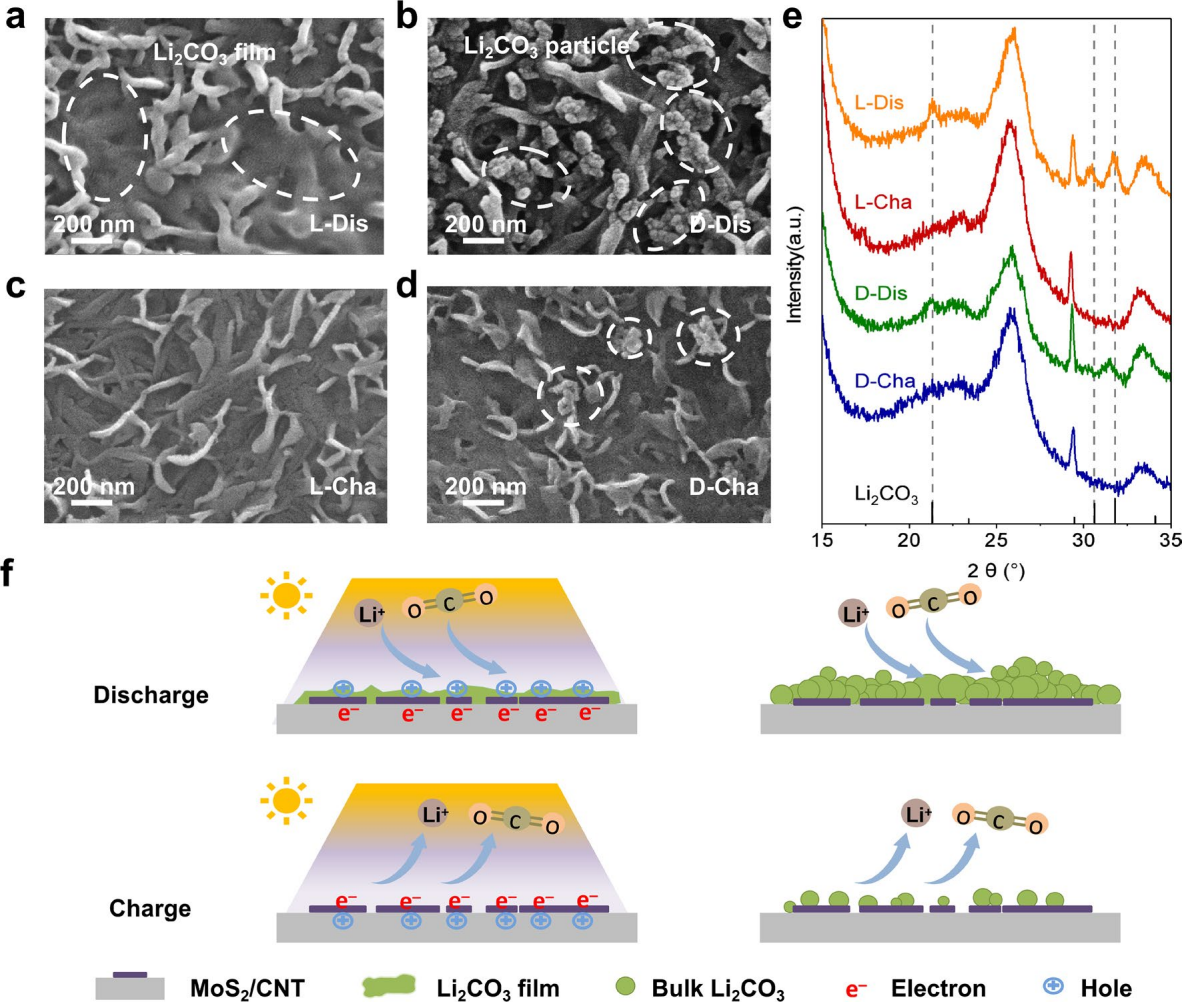
**a**–**d** Scanning electron microscopy images and **e** X-ray diffraction patterns of the MoS_2_/CNT cathodes collected from Li–CO_2_ batteries at corresponding discharge/charge states. L-Dis, L-Cha, D-Dis, and D-Cha represent the batteries discharged or recharged at 0.05 mAh cm^–2^ in the light or dark. **f** Model illustrating the effect of illumination on deposition and decomposition of discharge products in Li–CO_2_ batteries

The kinetic factors that vary with photoenergy affect the formation and decomposition of discharge product. To in-depth evaluate the effect of illumination on the morphology evolution of reaction product, the MoS_2_/CNT photocathodes after discharge and recharge at 0.05 mAh cm^–2^ with and without illumination were analyzed via SEM. Bulk discharge products formed by particles deposit on the surface of discharged MoS_2_/CNT cathode without illumination (Fig. 4b) and remain a small amount of residual after recharge with the same capacity (Fig. 4d). In sharp contrast, the light-mediated products exhibit film-like morphology which are mostly decomposed on recharged MoS_2_/CNT cathode. And a nearly clean cathode surface is delivered during the recharging process, suggesting the efficient catalytic performance of photo-generated carriers promote reversible decomposition of products (Fig. 4a, c). XRD characterization of MoS_2_/CNT was carried out to analyze the composition and evolution of the discharge products (Fig. 4e). Three characteristic peaks of Li_2_CO_3_ (2*θ* = 21.28°, 30.58°, and 31.68°) appear after discharge upon both illumination and no illumination [52]. After recharging process, the peaks corresponding to Li_2_CO_3_ disappear, confirming the reversible reaction in the Li–CO_2_ battery. To further measure the reversibility of Li–CO_2_ battery, differential quantitative mass spectrometry (DEMS) was performed to evaluate the CO_2_ conversion during the discharge and charge processes under a constant current density of 0.1 mA (Fig. S12) [53]. A discharge or charge capacity of 0.1 mAh corresponds to a theoretical CO_2_ evolution of 2.8 µmol. Under illumination, the consumption and release of CO_2_ were 2.37 and 2.07 µmol. Correspondingly, in the absence of light, the CO_2_ conversions during the discharge and charge processes were 2.20 and 1.54 µmol, respectively, indicating the superior ability of photoenergy on boosting decomposition of Li_2_CO_3_ and the reversibility of reaction.

A feasible mechanism for the tremendous difference in discharge product morphology is schematically clarified in Fig. 4f. Under illumination above the band gap energy, a large number of photoelectrons are excited from VB to CB in MoS_2_, delivering abundant available active sites for nucleation. Benefited from ample nucleation sites and fast-diffusing Li^+^, the Li_2_CO_3_ on photocathode grows more dispersive on surface and much slower in size than that in the dark. Therefore, Li_2_CO_3_ exhibited as thin film under light after discharge. During charging, sufficient photo-generated holes and better electronic transport of film-like morphology contribute to the decomposition of discharge Li_2_CO_3_, enabling the charge process at a much lower overpotential. On the contrary, bulk Li_2_CO_3_ formed by the accumulated particles grows on discharging cathode surface without illumination owing to slow Li^+^ spread and few nucleation sites. In the reverse charging process, bulk Li_2_CO_3_ decomposes difficultly due to the sluggish reaction kinetics caused by the absence of light, resulting in high overpotential. As cycle number increases, the accumulation of incompletely decomposing Li_2_CO_3_ hinders CO_2_ permeation and requires more energy for oxidation, following with higher and higher voltage gap and the loss of electrochemical performance.

### Electrochemical Performance of Room Temperature Li–CO_2_ Batteries

**Fig. 5:**
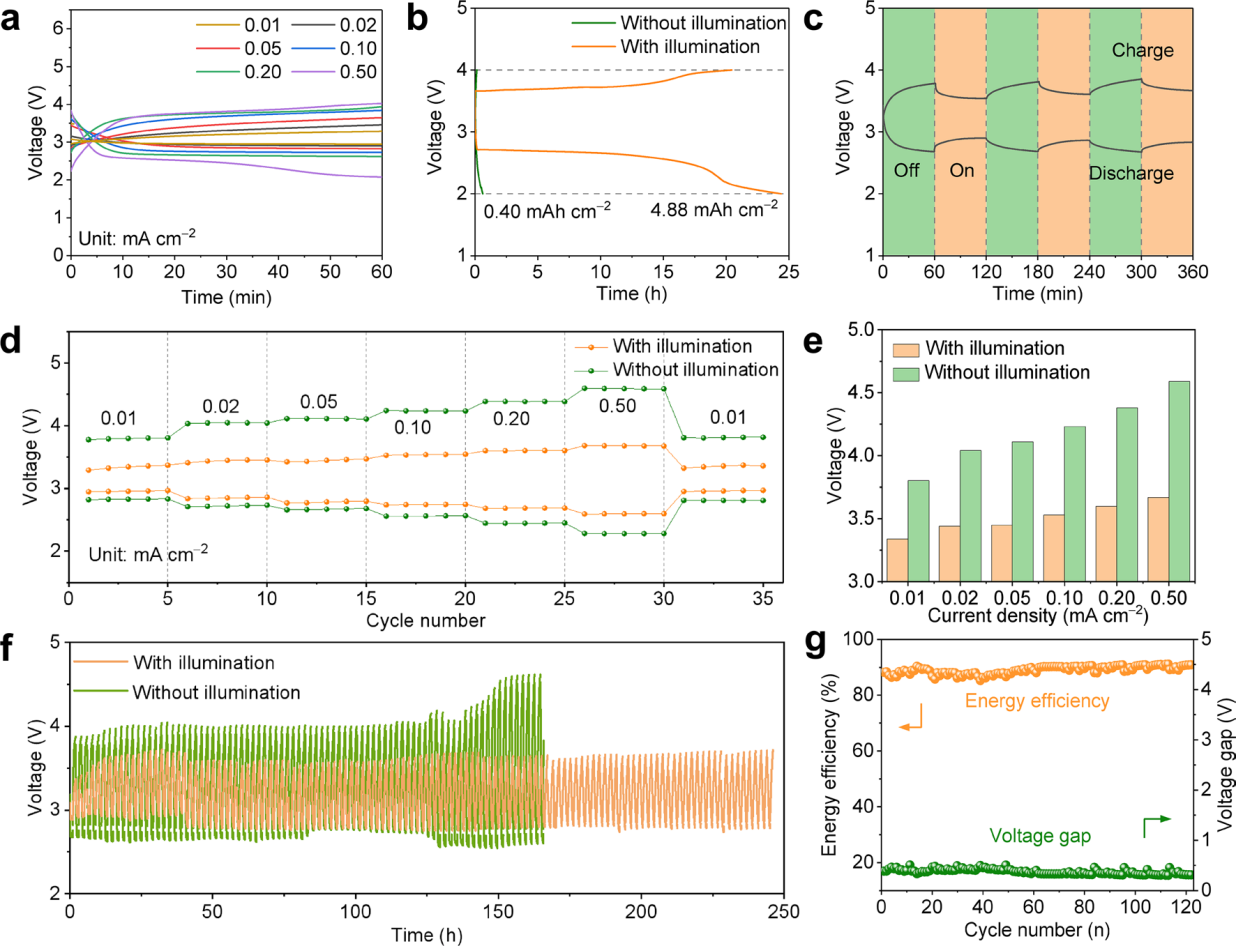
**a** Discharge and charge curves of the Li–CO_2_ battery based on the MoS_2_/CNT cathode with illumination at different current density. **b** Full discharge/charge profiles with cut-off voltages of 2 and 4 V. **c** Photo response of the discharge/charge voltage at a current density 0.01 mA cm^–2^ switching between "on" and "off". **d** Rate capability, **e** average charge terminal voltage at different current density, **f** cycling profiles at 0.02 mA cm^–1^ of Li–CO_2_ battery with MoS_2_/CNT cathode in the presence and absence of illumination, **g** energy efficiency and voltage gap of the battery

After confirming the photoelectric effect of the MoS_2_/CNT photocathode in electrochemical kinetics, the electrochemical properties of Li–CO_2_ battery were systematically evaluated. The discharge and charge profiles of at various current densities were performed in Figs. 5a and S13. Even with compensation of the photovoltage, similar to light-free condition, the polarization under illumination increased along with the current density, owning to the limitation of finite photo-generated carriers. At 0.02 mA cm^–2^, the overpotential under illumination rise to 0.55 V, which is 0.62 V lower than that of the battery without illumination. Similarly, owing to the compensative current, the polarization upon illumination increases along with the current density and rise to 0.91 V at 0.05 mA cm^–2^, which is 1.04 V lower than that of non-illuminated battery. As the current increases to 0.05, 0.10, 0.20, and 0.50 mA cm^–2^, the light-mitigated overpotential are 0.82, 1.11, 1.31, and 1.95 V, respectively. In sharp contrast, the charge voltage in the dark at 0.05 mA cm^–2^ hit the voltage cutoffs of 5 V, resulting in an extremely high voltage gap up to 2.64 V. With the increasing current density, Li–CO_2_ battery without illumination demonstrates more sharp polarization. The superior ion transport and electron conduction with photoenergy play a role at the electrochemical performance at different currents, resulting in lower overpotentials at the same capacity.

Meanwhile, fully discharged or charged with cut-off voltages of 2 or 4 V, the photo-energized battery provides high area capacities of 4.88 and 4.21 mAh cm^−2^, respectively, while the corresponding capacities of non-illuminated batteries are only 0.40 and 0.10 mAh cm^–2^ (Fig. 5b). The significant capacity increase is due to the great promotion on discharge performance of film-like Li_2_CO_3_ upon illumination. While the stacked bulk Li_2_CO_3_ is observed on completely discharged cathode without illumination, which is more difficult to decompose than that the film-like Li_2_CO_3_, as shown in Fig. S14. Furthermore, the photo-responsive voltage visually demonstrates the effect of solar energy on the potential during the discharge/charge process. As shown in Fig. 5c, the photo-responsive discharge voltage of the Li–CO_2_ battery with MoS_2_/CNT cathode rise from 2.68 to 2.90 V, and the charge voltage of 3.78 V rapidly decreases to 3.54 V. The sensitive and efficient photo-responsive voltage implies the sufficient generation and easy transport of photo-generated carriers in the MoS_2_/CNT cathode.

Figure 5d, e depicts the rate capability and corresponding average terminal potential at different current densities from 0.01 to 0.5 mA cm^–2^ at a fixed capacity of 0.1 mAh cm^–2^. In the entire current density range, the discharge voltages of the photo-treated cathode are higher than those in the dark and the charge voltages keep lower than the corresponding voltages of the non-illuminated cathode. Figure 5e visualizes the enhancement of overpotential gap in the dark or light with increasing current density during charging. When the current density is reduced to 0.01 mA cm^–2^, the voltage recovers to a value similar to that of the first five cycles, revealing the excellent reversibility inside the battery. The cycling performance of the battery was measured by galvanostatic discharge/charge at a current density of 0.02 mA cm^–2^. As shown in Fig. 5f, the light-mitigated discharge terminal voltage per cycle was consistently higher than that of the non-illuminated battery, resulting in the retention of lower overpotential and higher energy efficiency. After 120 cycles, the efficiency under light remains at 85% and polarization is very low (Fig. 5g). As shown in Fig. S15, the overall performance of the MoS_2_/CNT-based battery is superior to that of the previously reported photo-assisted Li–O_2_ and Li–CO_2_ batteries [54–59]. The efficiency reduction upon cycling is inferred to the accumulation of the volatilization of electrolyte during the long-term operation of the battery [60]. Correspondingly, the disparity in electrochemical properties can be well explained by the differences in kinetics and product morphology [61, 62]. The photo-enhanced ion transport, electron conduction, current density and active sites boost the formation and decomposition of discharge products, which are further manifested by superior overpotential, capacity and cycling performance in electrochemical properties.

### Electrochemical Performance of Low-Temperature Li–CO_2_ Batteries

**Fig. 6:**
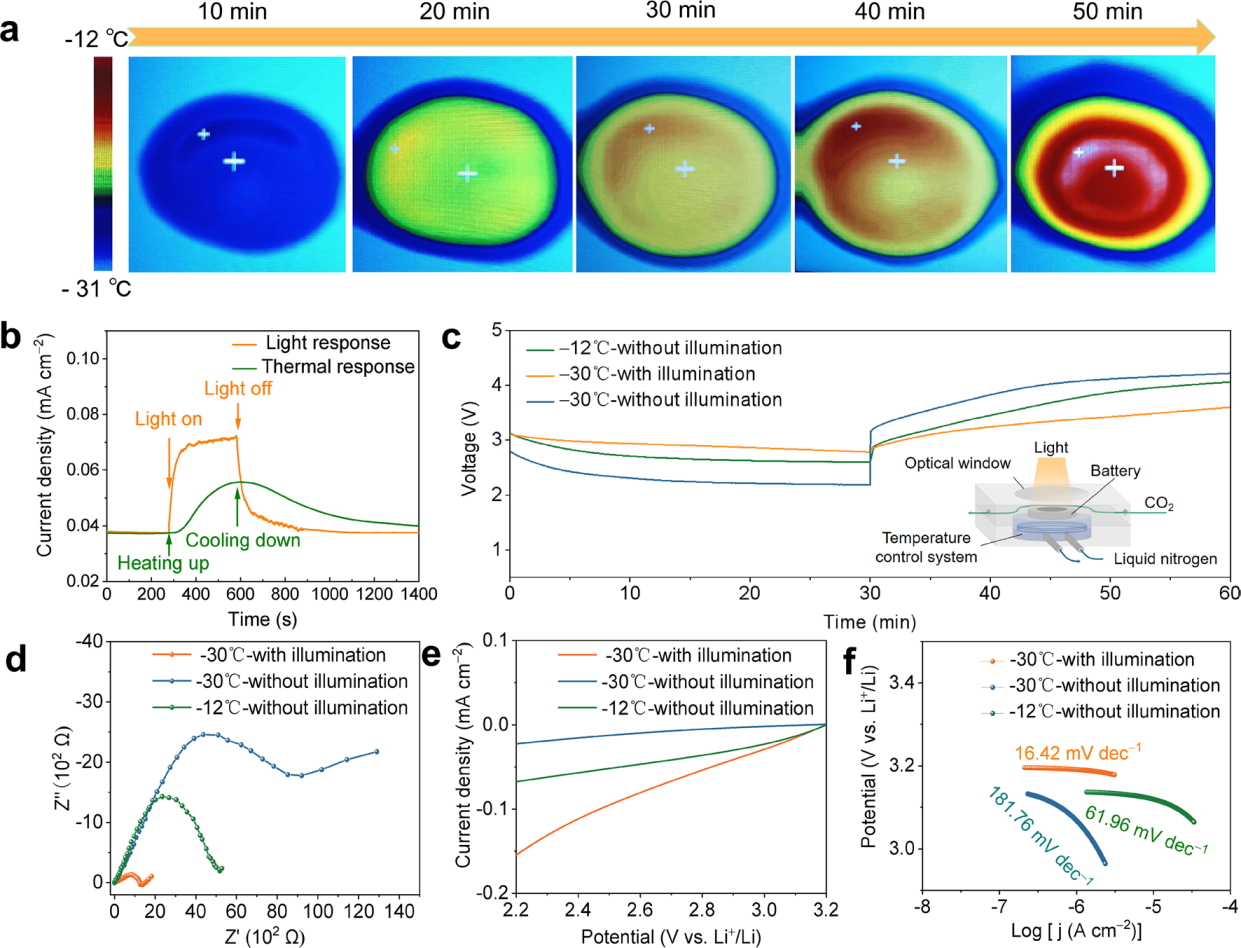
**a** IR images of top sides of MoS_2_/CNT cathode with illumination at − 30 °C. **b** On/off current response of MoS_2_/CNT under lighting (blue) and heating (orange) conditions at − 30 °C. **c** First discharge and charge curves, **d** electrochemical impedance spectroscopy spectra, **e** linear sweep voltammetry curves in CO_2_ reduction process at 5 mV s^–1^, and **f** corresponding Tafel curves of Li–CO_2_ battery with MoS_2_/CNT cathode with illumination at − 30 °C, without illumination at − 30 °C, and without illumination at − 12 °C

Based on the above results, the photoelectric effect of MoS_2_ effectively improves the electrochemical performance of room temperature Li–CO_2_ batteries, which has outdistanced the reported work [50, 63]. However, as shown in Fig. S16, the ionic conductivity of conventional electrolytes decreases drastically with decreasing temperature, which makes the kinetics of battery more sluggish at low temperatures. More importantly, photothermal effect of MoS_2_ can achieve extreme low-temperature Li–CO_2_ batteries by conversion of solar energy to heat. The low-temperature batteries were assembled in low-temperature control device without electrolyte replaced. To evaluate the photothermal conversion of the MoS_2_/CNT cathode under extreme conditions of − 30 °C, an infrared thermal imager (IR) was used to monitor the temperature of the cathode in real-time. By irradiating the electrode with a light source, the IR images are shown in Fig. 6a, it can be seen that MoS_2_/CNT gradually heats up with increasing irradiation time. After 50 min, the center temperature rises from − 30 to − 12 °C. Due to the continuous cooling of liquid nitrogen, there is no significant increase in temperature in the later stage. The IR image indicates the photothermal effect of MoS_2_/CNT under low-temperature conditions upon illumination, resulting in a temperature difference of approximately 18 °C. Based on the data obtained from IR images, the temperature gradient of the battery under − 30 °C illumination can be obtained. As shown in the Fig. S17, considering the thermal conductivity of the battery, the photo-energized low-temperature Li–CO_2_ battery based on MoS_2_/CNT cathode exhibits a temperature gradient of cathode-electrolyte-anode from − 12 to − 30 °C in this experimental environment.

To further explain the photoelectric and photothermal synergistic effects of MoS_2_/CNT at low temperatures, we compared the on/off light current response and up/cool thermal current response as shown in Fig. 6b. In the thermal current response curve (green line), the current density response delayed by 43 s after thermal excitation, and then reach the maximum platform after about 300 s. While MoS_2_/CNT warms from − 30 to − 12 °C within 10 s without illumination, the hysteresis of thermal response is limited by the heat conduction process. For the on/off photocurrent response of − 30 °C, the response of increased current density appears immediately within 1 s and quickly reaches the maximum platform, which indicating that photoelectric effect is far more sensitive than thermal response on the MoS_2_/CNT cathode. In addition, the low-temperature Li–CO_2_ battery shows a higher current platform than thermal effect, indicating that the MoS_2_/CNT in the presence of illumination has the photoelectric and photothermal synergistic, which makes the photo-energized Li–CO_2_ battery has catalytic performance than the thermal effect of environmental heating alone.

To confirm this assumption, we assembled a photo-energized Li–CO_2_ battery employing same electrolyte and Li foil as room temperature batteries, and compare electrochemical performance under three conditions with illumination at − 30 °C, without illumination at − 30 °C, and without illumination at − 12 °C. As shown in Fig. 6c, due to the slow reaction kinetics caused by low temperature in the − 30 °C, the Li–CO_2_ battery without illumination exhibits a low final discharge voltage of 2.18 V and a high final charge voltage of 4.22 V, resulting in an ultra-high over gap of 2.04 V. Under the ambient temperature of − 12 °C, the Li–CO_2_ battery without illumination exhibits higher the final discharge voltage of 2.58 V and lower charge voltage of 4.05 V, which indicate temperature dependence of reaction kinetics. The Li–CO_2_ battery with illumination at –30 °C further increases the final discharge voltage to 2.78 V and reduces the final charge voltage to 3.60 V, demonstrating a superior photothermal and photoelectric synergistic enhancement effect compared to the thermal effect under isothermal conditions. Figure S18 indicates that conventional electrolytes can also operate efficiently at low temperatures in the presence of light. The CV curves were further measured for the Li–CO_2_ battery to explore the catalytic performance at different temperature conditions. As is evident from the Fig. S19, the battery working at − 30 °C under illumination presents a lower onset evolution potential as well as a higher onset reduction potential along with significantly larger currents compared to the battery working at − 12 and − 30 °C without illumination. Moreover, Fig. S20 shows the discharge/charge behaviors responding to the Li–CO_2_ battery with illumination at − 30 °C and without illumination at − 12 °C. When the working environment is changed from illumination to only heating at − 12 °C, there is a rapid decrease in the discharge voltage, along with a quick increase of the charge voltage. This suggests both the discharge and charge processes of the Li–CO_2_ battery will be promoted by the photo-generated carriers.

Figure 6d shows the EIS of the Li–CO_2_ battery with illumination at − 30 °C, without illumination at − 30 °C, and without illumination at − 12 °C. The plot of EIS with illumination at − 30 °C shows a much smaller impedance of illuminated MoS_2_/CNT cathode than that under without illumination at − 12 and − 30 °C, which illustrates that photothermal and photoelectric synergistic accelerate the rapid ions diffuse and reaction kinetics at low temperature. In order to further investigate the effect of illumination, LSV curves of Li–CO_2_ batteries was evaluated. Under illumination at − 30 °C, the onset potentials for MoS_2_/CNT were found to be higher than those without illumination at − 12 and − 30 °C. For the CO_2_ reduction reaction in Fig. 6e, MoS_2_/CNT exhibits a higher current density than that under no illumination. And the Tafel slope inferred from the LSV data demonstrates that the photo-assisted value of 16.42 mV dec^−1^ is much smaller than that under no illumination at − 12 °C (61.96 mV dec^−1^) and −30 °C (181.76 mV dec^–1^) (Fig. 6f). For the opposite oxidation reaction, a similar result is observed in Fig. S21. The highest current density and the smallest Tafel slope with illumination at − 30 °C are attributed to enhanced dynamic kinetics and conductivity during discharge and charge process. As a result, the Li–CO_2_ battery under illumination at − 30 °C shows smaller over gap during 10 cycles compared to the battery without illumination at − 12 °C as shown in Fig. S22, indicating that the photo-energized effect can superimpose the photoelectric effect on the thermal effect, further improving the cycling performance of low-temperature Li–CO_2_ batteries. Besides, the rate capability of the Li–CO_2_ battery under illumination at − 30 °C in Fig. S23 exhibits smaller polarization with increasing current density than the battery without illumination at − 12 °C.

## Conclusions

In summary, we successfully developed a photo-energized Li–CO_2_ battery based on MoS_2_/CNT photo-electrode as cathode in wide temperature range application. The binder-free structure of MoS_2_/CNT cathode enables abundant generation and rapid transfer of photo-excited carriers, which facilitates the intrinsic dynamic kinetics. Upon illumination, photo-generated electrons transiting from VB to CB migrate to participate in the reduction of CO_2_, leading to different morphology during discharge process. During reverse charge process, photo-generated holes have a favorable impact on the decomposition of insulated discharge products Li_2_CO_3_. Consequently, the photo-energized room temperature battery exhibits a higher discharge voltage platform of 2.95 V and the charge voltage down to 3.27 V, leading to high energy efficiency of 90.2% than 74.9% of non-illuminated battery. And excellent cycling stability indicates the conversion and compensation of photoenergy for electrochemical reaction. Toward extreme low temperature, the highly performance Li–CO_2_ batteries profit from the photoelectric and photothermal synergistic mechanism of MoS_2_/CNT cathode, achieving an ultra-low median charge voltage of 3.4 V at − 30 °C with a round-trip efficiency of 86.6%. These results propose useful guidelines for MoS_2_ as photocathode in performance enhancement of photo-energized Li–CO_2_ systems in a wide temperature range for energy storage.

## Supplementary Information

Below is the link to the electronic supplementary material.

Supplementary file1 (DOCX 2542 kb)

## References

[1] C.T. Dinh, T. Burdyny, M.G. Kibria, A. Seifitokaldani, C.M. Gabardo et al., CO_2_ electroreduction to ethylene via hydroxide-mediated copper catalysis at an abrupt interface. Science **360**, 783–787 (2018). 10.1126/science.aas9100 29773749 10.1126/science.aas9100

[2] Z. Zhuo, K. Dai, R. Qiao, R. Wang, J. Wu et al., Cycling mechanism of Li_2_MnO_3_: Li–CO_2_ batteries and commonality on oxygen redox in cathode materials. Joule **5**, 975–997 (2021). 10.1016/j.joule.2021.02.004

[3] H.-D. Lim, B. Lee, Y. Zheng, J. Hong, J. Kim et al., Rational design of redox mediators for advanced Li–O_2_ batteries. Nat. Energy **1**, 16066 (2016). 10.1038/nenergy.2016.66

[4] S.-M. Xu, Z.-C. Ren, X. Liu, X. Liang, K.-X. Wang et al., Carbonate decomposition: low-overpotential Li–CO_2_ battery based on interlayer-confined monodisperse catalyst. Energy Storage Mater. **15**, 291–298 (2018). 10.1016/j.ensm.2018.05.015

[5] B. Liu, Y. Sun, L. Liu, J. Chen, B. Yang et al., Recent advances in understanding Li–CO_2_ electrochemistry. Energy Environ. Sci. **12**, 887–922 (2019). 10.1039/c8ee03417f

[6] Y. Xu, X. Li, Y. Li, Y. Wang, L. Song et al., Reconfiguration of the charge density difference of nitrogen-doped graphene by covalently bonded Cu-N_4_ active sites boosting thermodynamics and performance in aprotic Li-CO_2_ battery. Energy Storage Mater. **68**, 103354 (2024). 10.1016/j.ensm.2024.103354

[7] X. Chen, Y. Zhang, C. Chen, H. Li, Y. Lin et al., Atomically dispersed ruthenium catalysts with open hollow structure for lithium–oxygen batteries. Nano-Micro Lett. **16**, 27 (2023). 10.1007/s40820-023-01240-0 10.1007/s40820-023-01240-0 PMC10663429 37989893

[8] Z. Ye, Y. Jiang, L. Li, F. Wu, R. Chen, Rational design of MOF-based materials for next-generation rechargeable batteries. Nano-Micro Lett. **13**, 203 (2021). 10.1007/s40820-021-00726-z 10.1007/s40820-021-00726-z PMC8492800 34611765

[9] C. Li, Y. Ji, Y. Wang, C. Liu, Z. Chen et al., Applications of metal-organic frameworks and their derivatives in electrochemical CO_2_ reduction. Nano-Micro Lett. **15**, 113 (2023). 10.1007/s40820-023-01092-8 10.1007/s40820-023-01092-8 PMC10149437 37121938

[10] L. Qie, Y. Lin, J.W. Connell, J. Xu, L. Dai, Highly rechargeable lithium-CO_2_ batteries with a boron- and nitrogen-codoped holey-graphene cathode. Angew. Chem. Int. Ed. **56**, 6970–6974 (2017). 10.1002/anie.201701826 10.1002/anie.201701826 28510337

[11] C. Wang, Q. Zhang, X. Zhang, X.-G. Wang, Z. Xie et al., Fabricating Ir/C nanofiber networks as free-standing air cathodes for rechargeable Li–CO_2_ batteries. Small **14**, e1800641 (2018). 10.1002/smll.201800641 29882379 10.1002/smll.201800641

[12] X. Mu, H. Pan, P. He, H. Zhou, Li–CO_2_ and Na–CO_2_ batteries: toward greener and sustainable electrical energy storage. Adv. Mater. **32**, 1903790 (2020). 10.1002/adma.201903790 10.1002/adma.201903790 31512290

[13] Y. Li, J. Zhou, T. Zhang, T. Wang, X. Li et al., Highly surface-wrinkled and N-doped CNTs anchored on metal wire: a novel fiber-shaped cathode toward high-performance flexible Li–CO_2_ batteries. Adv. Funct. Mater. **29**, 1808117 (2019). 10.1002/adfm.201808117

[14] P.-F. Zhang, J.-Y. Zhang, T. Sheng, Y.-Q. Lu, Z.-W. Yin et al., Synergetic effect of Ru and NiO in the electrocatalytic decomposition of Li_2_CO_3_ to enhance the performance of a Li–CO_2_/O_2_ battery. ACS Catal. **10**, 1640–1651 (2020). 10.1021/acscatal.9b04138

[15] J. Sun, Y. Lu, H. Yang, M. Han, L. Shao et al., Rechargeable Na–CO_2_ batteries starting from cathode of Na_2_CO_3_ and carbon nanotubes. Research **2018**, 6914626 (2018). 10.1155/2018/6914626 31549035 10.1155/2018/6914626 PMC6750045

[16] W. Ma, S. Lu, X. Lei, X. Liu, Y. Ding, Porous Mn_2_O_3_ cathode for highly durable Li–CO_2_ batteries. J. Mater. Chem. A **6**, 20829–20835 (2018). 10.1039/c8ta06143b

[17] Z. Lian, Y. Lu, C. Wang, X. Zhu, S. Ma et al., Single-atom Ru implanted on Co_3_O_4_ nanosheets as efficient dual-catalyst for Li–CO_2_ batteries. Adv. Sci. **8**, e2102550 (2021). 10.1002/advs.202102550 10.1002/advs.202102550 PMC8655220 34672110

[18] L. Fei, Y. Yin, M. Yang, S. Zhang, C. Wang, Wearable solar energy management based on visible solar thermal energy storage for full solar spectrum utilization. Energy Storage Mater. **42**, 636–644 (2021). 10.1016/j.ensm.2021.07.049

[19] W. Feng, L. Zhu, X. Dong, Y. Wang, Y. Xia et al., Enhanced moisture stability of lithium-rich antiperovskites for sustainable all-solid-state lithium batteries. Adv. Mater. **35**, e2210365 (2023). 10.1002/adma.202210365 36583712 10.1002/adma.202210365

[20] T. Fang, H. Huang, J. Feng, Y. Hu, Q. Qian et al., Reactive inorganic vapor deposition of perovskite oxynitride films for solar energy conversion. Research **2019**, 9282674 (2019). 10.34133/2019/9282674 31922145 10.34133/2019/9282674 PMC6946269

[21] Q. Guo, J. Wu, Y. Yang, X. Liu, Z. Lan et al., High-performance and hysteresis-free perovskite solar cells based on rare-earth-doped SnO_2_ mesoporous scaffold. Research **2019**, 4049793 (2019). 10.34133/2019/4049793 31912035 10.34133/2019/4049793 PMC6944519

[22] J. Wu, Y. Huang, W. Ye, Y. Li, CO_2_ reduction: from the electrochemical to photochemical approach. Adv. Sci. **4**, 1700194 (2017). 10.1002/advs.201700194 10.1002/advs.201700194 PMC5700640 29201614

[23] F. Podjaski, J. Kröger, B.V. Lotsch, Toward an aqueous solar battery: direct electrochemical storage of solar energy in carbon nitrides. Adv. Mater. **30**, 1705477 (2018). 10.1002/adma.201705477 10.1002/adma.201705477 29318675

[24] L. Xu, Y. Ren, Y. Fu, M. Liu, F. Zhu et al., Strong photo-thermal coupling effect boosts CO_2_ reduction into CH_4_ in a concentrated solar reactor. Chem. Eng. J. **468**, 143831 (2023). 10.1016/j.cej.2023.143831

[25] S. Xu, C. Chen, Y. Kuang, J. Song, W. Gan et al., Flexible lithium–CO_2_ battery with ultrahigh capacity and stable cycling. Energy Environ. Sci. **11**, 3231–3237 (2018). 10.1039/c8ee01468j

[26] K. Baek, W.C. Jeon, S. Woo, J.C. Kim, J.G. Lee et al., Synergistic effect of quinary molten salts and ruthenium catalyst for high-power-density lithium-carbon dioxide cell. Nat. Commun. **11**, 456 (2020). 10.1038/s41467-019-14121-1 31974360 10.1038/s41467-019-14121-1 PMC6978343

[27] K.M. Naik, A.K. Chourasia, M. Shavez, C.S. Sharma, Bimetallic RuNi electrocatalyst coated MWCNTs cathode for an efficient and stable Li–CO_2_ and Li–CO_2 Mars_ batteries performance with low overpotential. Chemsuschem **16**, e202300734 (2023). 10.1002/cssc.202300734 37317946 10.1002/cssc.202300734

[28] J.-H. Kang, J. Park, M. Na, R.H. Choi, H.R. Byon, Low-temperature CO_2_-assisted lithium–oxygen batteries for improved stability of peroxodicarbonate and excellent cyclability. ACS Energy Lett. **7**, 4248–4257 (2022). 10.1021/acsenergylett.2c01796

[29] W. Cui, C. Ma, X. Lei, Y. Lv, Q. Zhang et al., Gel electrolyte with dimethyl sulfoxide confined in a polymer matrix for Li-air batteries operable at sub-zero temperature. J. Power. Sources **577**, 233264 (2023). 10.1016/j.jpowsour.2023.233264

[30] H. Kim, J.Y. Hwang, Y.G. Ham, H.N. Choi, M.H. Alfaruqi et al., Turning on lithium-sulfur full batteries at -10 °C. ACS Nano **17**, 14032–14042 (2023). 10.1021/acsnano.3c04213 37428961 10.1021/acsnano.3c04213

[31] A. Gupta, A. Manthiram, Designing advanced lithium-based batteries for low-temperature conditions. Adv. Energy Mater. **10**, 2001972 (2020). 10.1002/aenm.202001972 34158810 10.1002/aenm.202001972 PMC8216142

[32] J. Li, L. Wang, Y. Zhao, S. Li, X. Fu et al., Li–CO_2_ batteries efficiently working at ultra-low temperatures. Adv. Funct. Mater. **30**, 2001619 (2020). 10.1002/adfm.202001619

[33] D.-H. Guan, X.-X. Wang, F. Li, L.-J. Zheng, M.-L. Li et al., All-solid-state photo-assisted Li–CO_2_ battery working at an ultra-wide operation temperature. ACS Nano **16**, 12364–12376 (2022). 10.1021/acsnano.2c03534 35914235 10.1021/acsnano.2c03534

[34] D. Zhu, Q. Zhao, G. Fan, S. Zhao, L. Wang et al., Photoinduced oxygen reduction reaction boosts the output voltage of a zinc-air battery. Angew. Chem. Int. Ed. **58**, 12460–12464 (2019). 10.1002/anie.201905954 10.1002/anie.201905954 31273902

[35] M. Li, X. Wang, F. Li, L. Zheng, J. Xu et al., A bifunctional photo-assisted Li–O_2_ battery based on a hierarchical heterostructured cathode. Adv. Mater. **32**, e1907098 (2020). 10.1002/adma.201907098 32671896 10.1002/adma.201907098

[36] H. Song, S. Wang, X. Song, J. Wang, K. Jiang et al., Solar-driven all-solid-state lithium–air batteries operating at extreme low temperatures. Energy Environ. Sci. **13**, 1205–1211 (2020). 10.1039/c9ee04039k

[37] H. Zhang, J. Luo, M. Qi, S. Lin, Q. Dong et al., Enabling lithium metal anode in nonflammable phosphate electrolyte with electrochemically induced chemical reactions. Angew. Chem. Int. Ed. **60**, 19183–19190 (2021). 10.1002/anie.202103909 10.1002/anie.202103909 33928733

[38] G.M. Carroll, H. Zhang, J.R. Dunklin, E.M. Miller, N.R. Neale et al., Unique interfacial thermodynamics of few-layer 2D MoS_2_ for (photo)electrochemical catalysis. Energy Environ. Sci. **12**, 1648–1656 (2019). 10.1039/c9ee00513g

[39] S. Song, Z. Xing, K. Wang, H. Zhao, P. Chen et al., 3D flower-like mesoporous Bi_4_O_5_I_2_/MoS_2_ Z-scheme heterojunction with optimized photothermal-photocatalytic performance. Green Energy Environ. **8**, 200–212 (2023). 10.1016/j.gee.2021.03.013

[40] Y. Liu, R. Wang, Y. Lyu, H. Li, L. Chen, Rechargeable Li/CO_2_–O_2_ (2: 1) battery and Li/CO_2_ battery. Energy Environ. Sci. **7**, 677–681 (2014). 10.1039/C3EE43318H

[41] J. Wang, W. Fang, Y. Hu, Y. Zhang, J. Dang et al., Single atom Ru doping 2H-MoS_2_ as highly efficient hydrogen evolution reaction electrocatalyst in a wide pH range. Appl. Catal. B Environ. **298**, 120490 (2021). 10.1016/j.apcatb.2021.120490

[42] H.-Y. Lin, K.T. Le, P.-H. Chen, J.M. Wu, Systematic investigation of the piezocatalysis–adsorption duality of polymorphic MoS_2_ nanoflowers. Appl. Catal. B Environ. **317**, 121717 (2022). 10.1016/j.apcatb.2022.121717

[43] X. Gan, H. Zhao, D. Lei, P. Wang, Improving electrocatalytic activity of 2H-MoS_2_ nanosheets obtained by liquid phase exfoliation: Covalent surface modification versus interlayer interaction. J. Catal. **391**, 424–434 (2020). 10.1016/j.jcat.2020.09.009

[44] P. Tiwari, D. Janas, R. Chandra, Self-standing MoS_2_/CNT and MnO_2_/CNT one dimensional core shell heterostructures for asymmetric supercapacitor application. Carbon **177**, 291–303 (2021). 10.1016/j.carbon.2021.02.080

[45] H. Wang, X. Xu, A. Neville, Facile synthesis of vacancy-induced 2H-MoS_2_ nanosheets and defect investigation for supercapacitor application. RSC Adv. **11**, 26273–26283 (2021). 10.1039/D1RA04902J 35479470 10.1039/d1ra04902j PMC9037448

[46] L.X. Chen, Z.W. Chen, Y. Wang, C.C. Yang, Q. Jiang, Design of dual-modified MoS_2_ with nanoporous Ni and graphene as efficient catalysts for the hydrogen evolution reaction. ACS Catal. **8**, 8107–8114 (2018). 10.1021/acscatal.8b01164

[47] R. Meng, F. Li, D. Li, B. Jin, A green and efficient synthesis method of Benzo[c]cinnolines: electrochemical reduction of 2, 2’-Dinitrobiphenyl in the presence of CO_2_. ChemElectroChem **9**, 2101381 (2022). 10.1002/celc.202101381

[48] D. Sun, D. Huang, H. Wang, G.-L. Xu, X. Zhang et al., 1T MoS_2_ nanosheets with extraordinary sodium storage properties via thermal-driven ion intercalation assisted exfoliation of bulky MoS_2_. Nano Energy **61**, 361–369 (2019). 10.1016/j.nanoen.2019.04.063

[49] Z. Lu, M. Xiao, S. Wang, D. Han, Z. Huang et al., Correction: a rechargeable Li–CO_2_ battery based on the preservation of dimethyl sulfoxide. J. Mater. Chem. A **10**, 15839 (2022). 10.1039/d2ta02586h

[50] Z. Zhu, X. Shi, G. Fan, F. Li, J. Chen, Photo-energy conversion and storage in an aprotic Li–O_2_ battery. Angew. Chem. Int. Ed. **58**, 19021–19026 (2019). 10.1002/anie.201911228 10.1002/anie.201911228 31591805

[51] D.-H. Guan, X.-X. Wang, M.-L. Li, F. Li, L.-J. Zheng et al., Light/electricity energy conversion and storage for a hierarchical porous In_2_S_3_@CNT/SS cathode towards a flexible Li–CO_2_ battery. Angew. Chem. Int. Ed. **59**, 19518–19524 (2020). 10.1002/anie.202005053 10.1002/anie.202005053 32419313

[52] Z. Wang, B. Liu, X. Yang, C. Zhao, P. Dong et al., Dual catalytic sites of alloying effect bloom CO_2_ catalytic conversion for highly stable Li–CO_2_ battery. Adv. Funct. Mater. **33**, 2213931 (2023). 10.1002/adfm.202213931

[53] X. Sun, X. Mu, W. Zheng, L. Wang, S. Yang et al., Binuclear Cu complex catalysis enabling Li–CO_2_ battery with a high discharge voltage above 3.0 V. Nat. Commun. **14**, 536 (2023). 10.1038/s41467-023-36276-8 36725869 10.1038/s41467-023-36276-8 PMC9892515

[54] X. Yu, H. Gong, B. Gao, X. Fan, P. Li et al., Illumination-enhanced oxygen reduction kinetics in hybrid lithium-oxygen battery with p-type semiconductor. Chem. Eng. J. **449**, 137774 (2022). 10.1016/j.cej.2022.137774

[55] H. Gong, T. Wang, K. Chang, P. Li, L. Liu et al., Revealing the illumination effect on the discharge products in high-performance Li–O_2_ batteries with heterostructured photocatalysts. Carbon Energy **4**, 1169–1181 (2022). 10.1002/cey2.208

[56] K. Zhang, J. Li, W. Zhai, C. Li, Z. Zhu et al., Boosting cycling stability and rate capability of Li–CO_2_ batteries via synergistic photoelectric effect and plasmonic interaction. Angew. Chem. Int. Ed. **61**, e202201718 (2022). 10.1002/anie.202201718 10.1002/anie.202201718 35192236

[57] X.-X. Wang, D.-H. Guan, F. Li, M.-L. Li, L.-J. Zheng et al., A renewable light-promoted flexible Li–CO_2_ battery with ultrahigh energy efficiency of 97.9%. Small **17**, e2100642 (2021). 10.1002/smll.202100642 34081392 10.1002/smll.202100642

[58] J.-N. Chang, S. Li, Q. Li, J.-H. Wang, C. Guo et al., Redox molecular junction metal-covalent organic frameworks for light-assisted CO_2_ energy storage. Angew. Chem. Int. Ed. **63**, e202402458 (2024). 10.1002/anie.202402458 10.1002/anie.202402458 38545814

[59] Z. Li, M.-L. Li, X.-X. Wang, D.-H. Guan, W.-Q. Liu et al., In situ fabricated photo-electro-catalytic hybrid cathode for light-assisted lithium–CO_2_ batteries. J. Mater. Chem. A **8**, 14799–14806 (2020). 10.1039/d0ta05069e

[60] S. Chen, H. Wang, S. Lu, Y. Xiang, Monolayer MoS_2_ film supported metal electrocatalysts: a DFT study. RSC Adv. **6**, 107836–107839 (2016). 10.1039/C6RA23995A

[61] Y. Bae, H. Song, H. Park, H.-D. Lim, H.J. Kwon et al., Dual-functioning molecular carrier of superoxide radicals for stable and efficient lithium–oxygen batteries. Adv. Energy Mater. **10**, 1904187 (2020). 10.1002/aenm.201904187

[62] H. Gong, X. Yu, Y. Xu, B. Gao, H. Xue et al., Long-life reversible Li-CO_2_ batteries with optimized Li_2_CO_3_ flakes as discharge products on palladium-copper nanoparticles. Inorg. Chem. Front. **9**, 1533–1540 (2022). 10.1039/D1QI01583D

[63] A. Ahmadiparidari, R.E. Warburton, L. Majidi, M. Asadi, A. Chamaani et al., A long-cycle-life lithium-CO_2_ battery with carbon neutrality. Adv. Mater. **31**, e1902518 (2019). 10.1002/adma.201902518 31441124 10.1002/adma.201902518

