# Supplementary material for: Photo-Energized MoS2/CNT Cathode for High-Performance Li–CO2 Batteries in a Wide-Temperature Range
Source: Nanomicro Lett. 2024 Sep 21;17:5. doi: 10.1007/s40820-024-01506-1 (PMC11415333; doi:10.1007/s40820-024-01506-1)
Supplement: Supplementary file 1 — Supplementary file1 (DOCX 2542 kb) [file 40820_2024_1506_MOESM1_ESM.docx]

Supporting Information for

**Photo-Energized MoS_2_/CNT Cathode for High Performance Li–CO_2_ Batteries in a Wide Temperature Range**

Tingsong Hu^1^, Wenyi Lian^1^, Kang Hu^1^, Qiuju Li^1^, Xueliang Cui^1^, Tengyu Yao^1^, Laifa Shen^1,^*

^1^Jiangsu Key Laboratory of Materials and Technologies for Energy Storage, College of Materials Science and Technology, Nanjing University of Aeronautics and Astronautics, Nanjing 210016, People’s Republic of China.

*Corresponding author. E-mail: [lfshen@nuaa.edu.cn](mailto:lfshen@nuaa.edu.cn) (Laifa Shen)

**Supplementary Fiures and Tables**


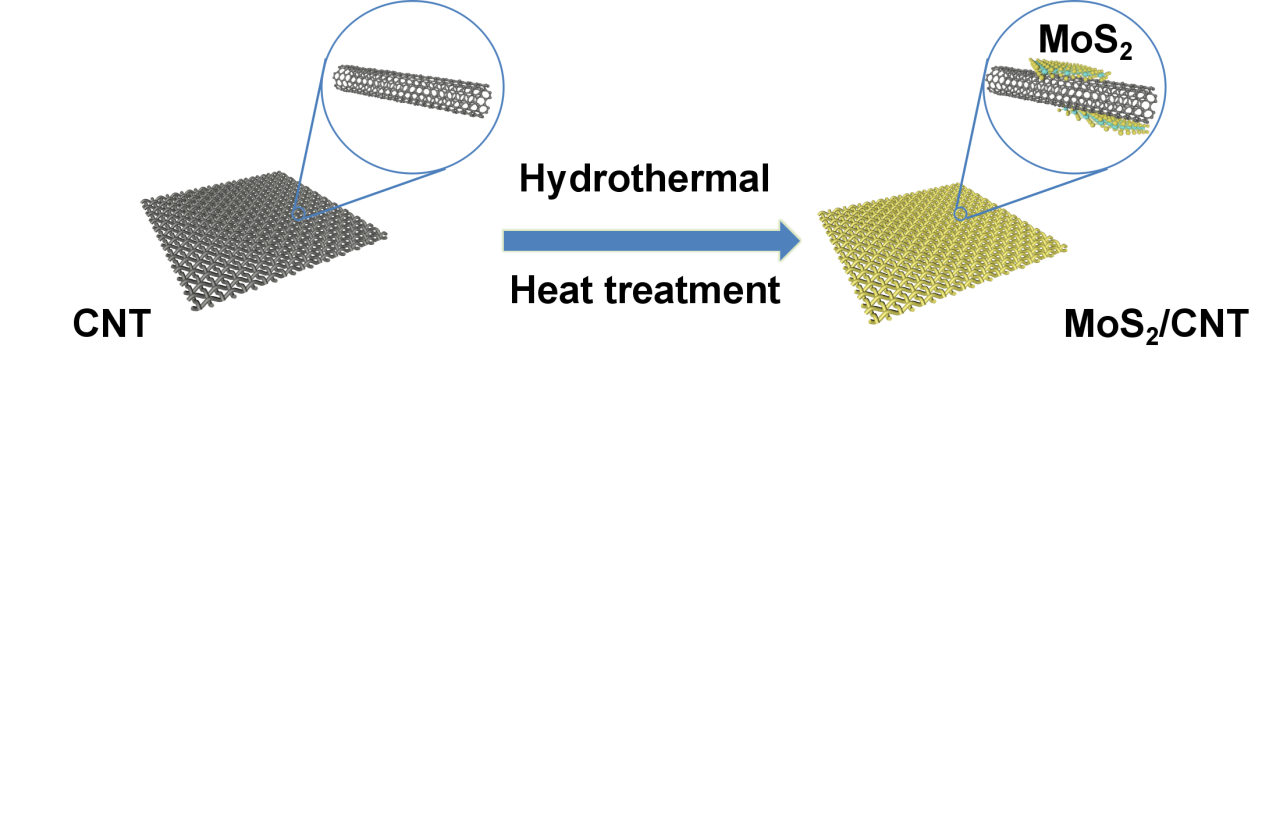


**Fig. S1** Representations of the design of MoS_2_/CNT


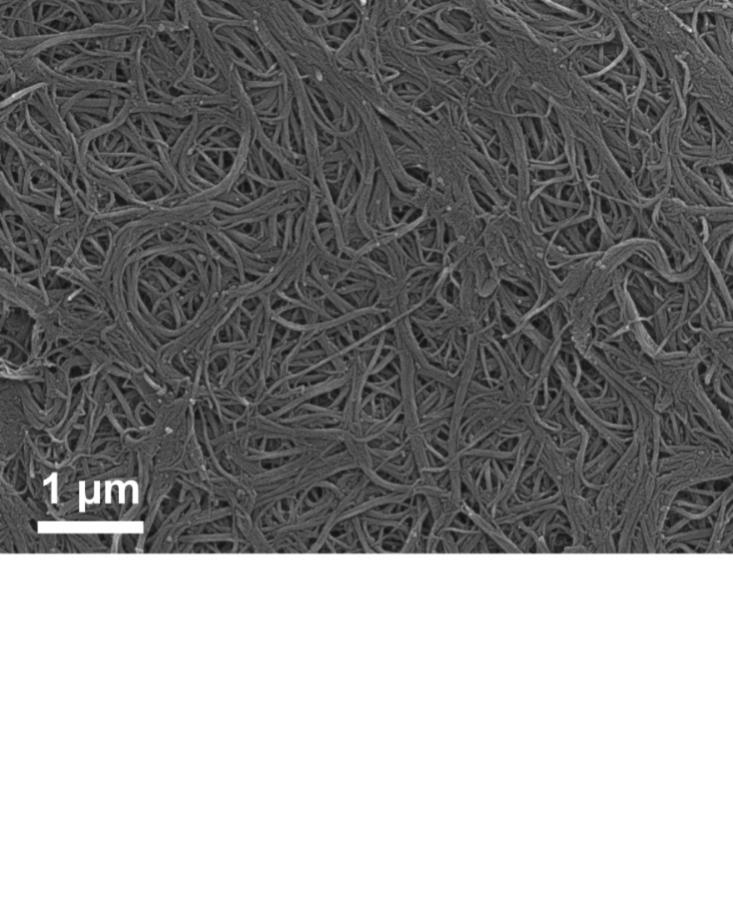


**Fig. S2** Scanning electron microscopy image of pristine CNT


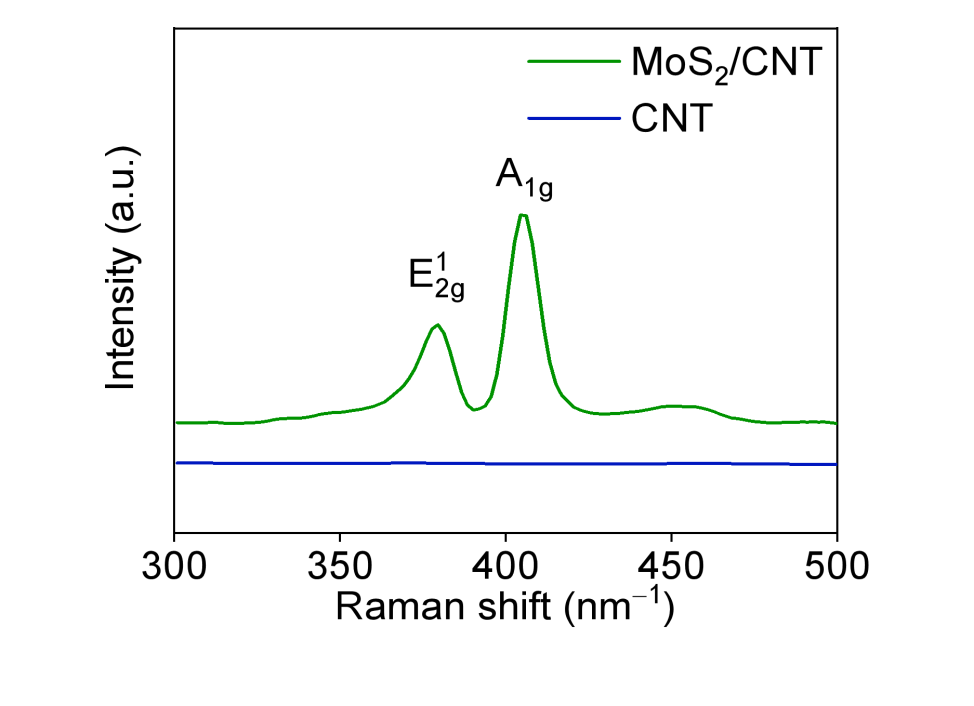


**Fig. S3** Raman spectra of CNT and MoS_2_/CNT cathode


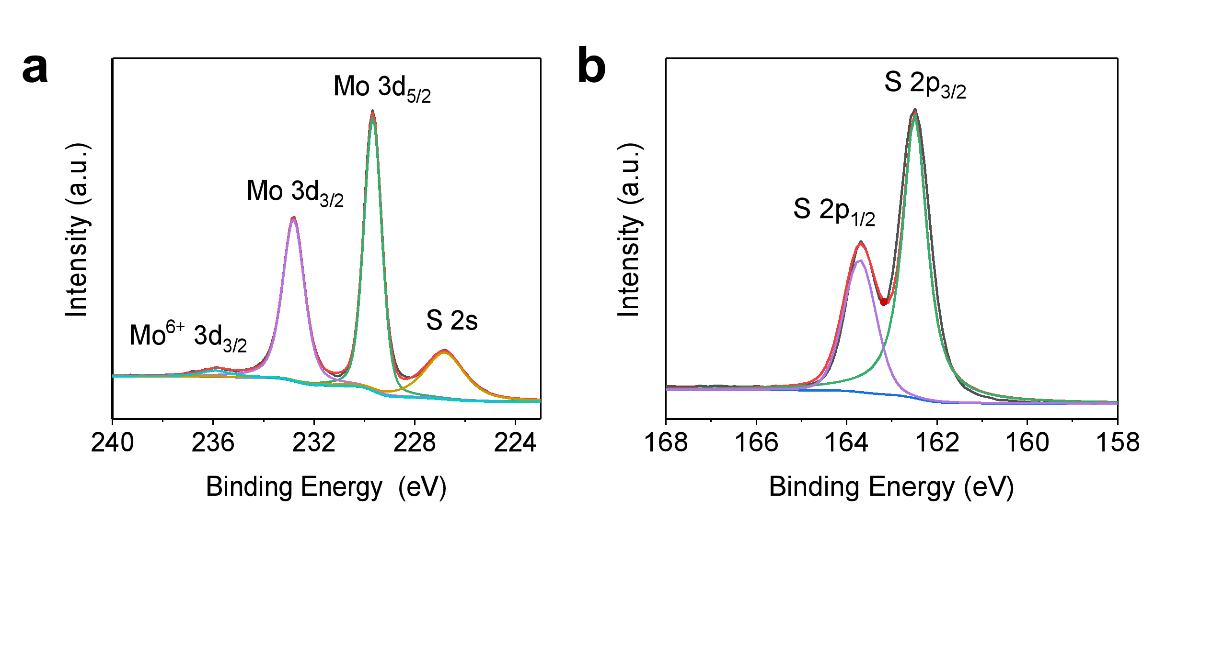


**Fig. S4** X-ray photoelectron spectroscopy image of (**a**) Mo and (**b**) S in MoS_2_/CNT cathode


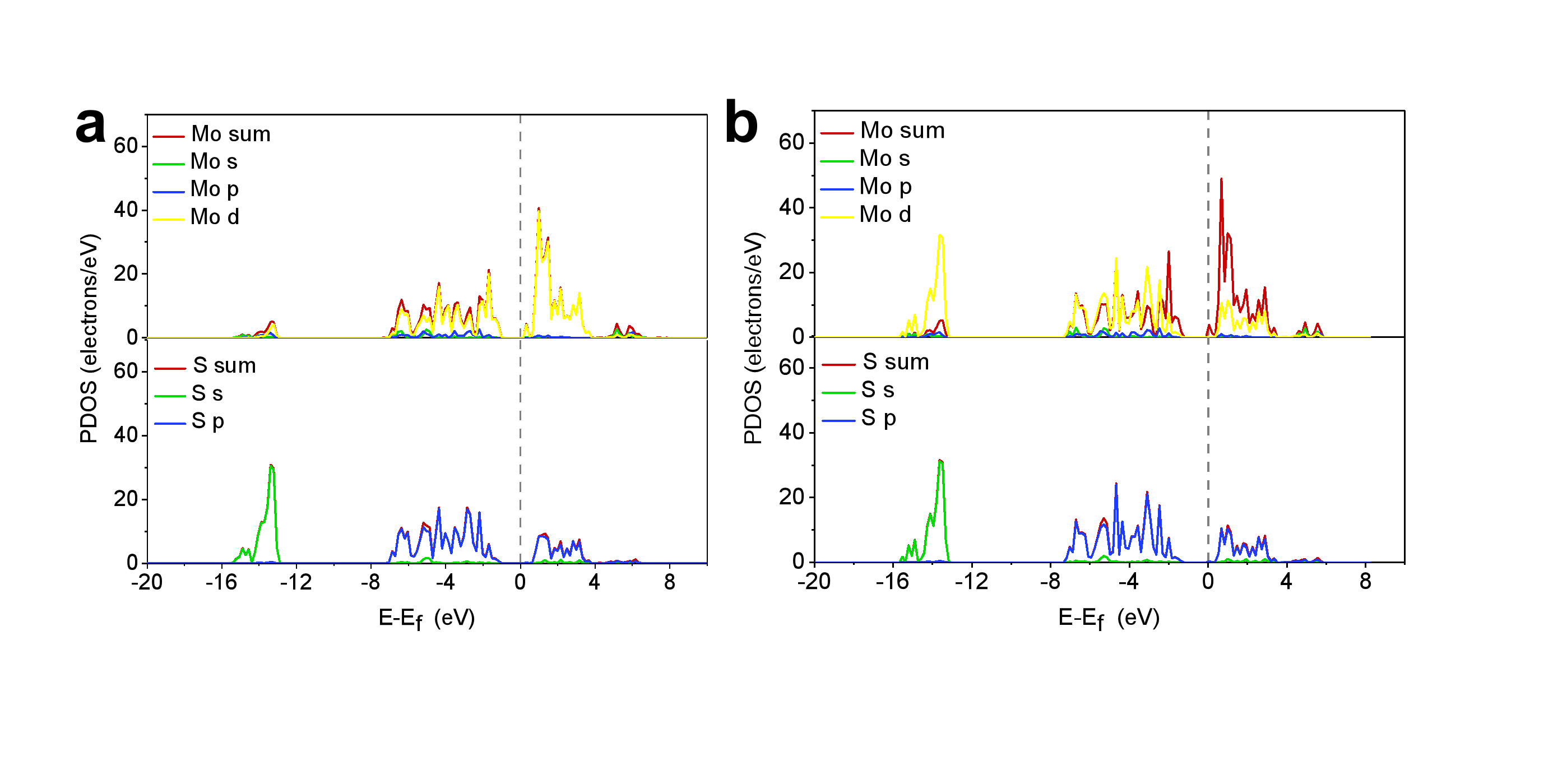


**Fig. S5** Partial Density of States of Mo and S in (**a**) MoS_2_/CNT or (**b**) MoS_2_


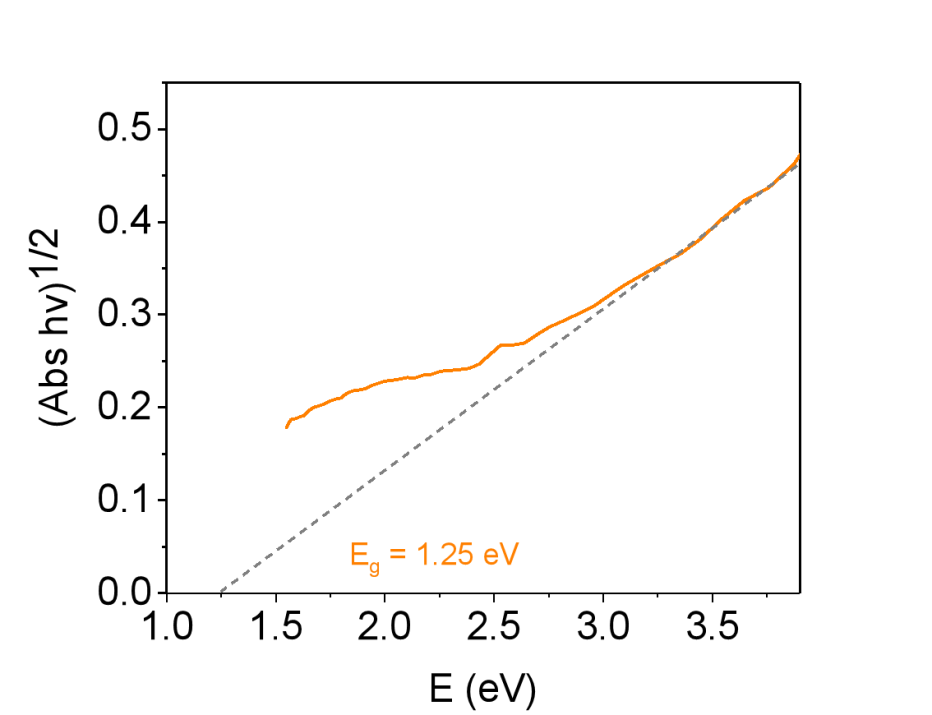


**Fig. S6** Tauc plot corresponding to UV-vis absorption of MoS_2_/CNT


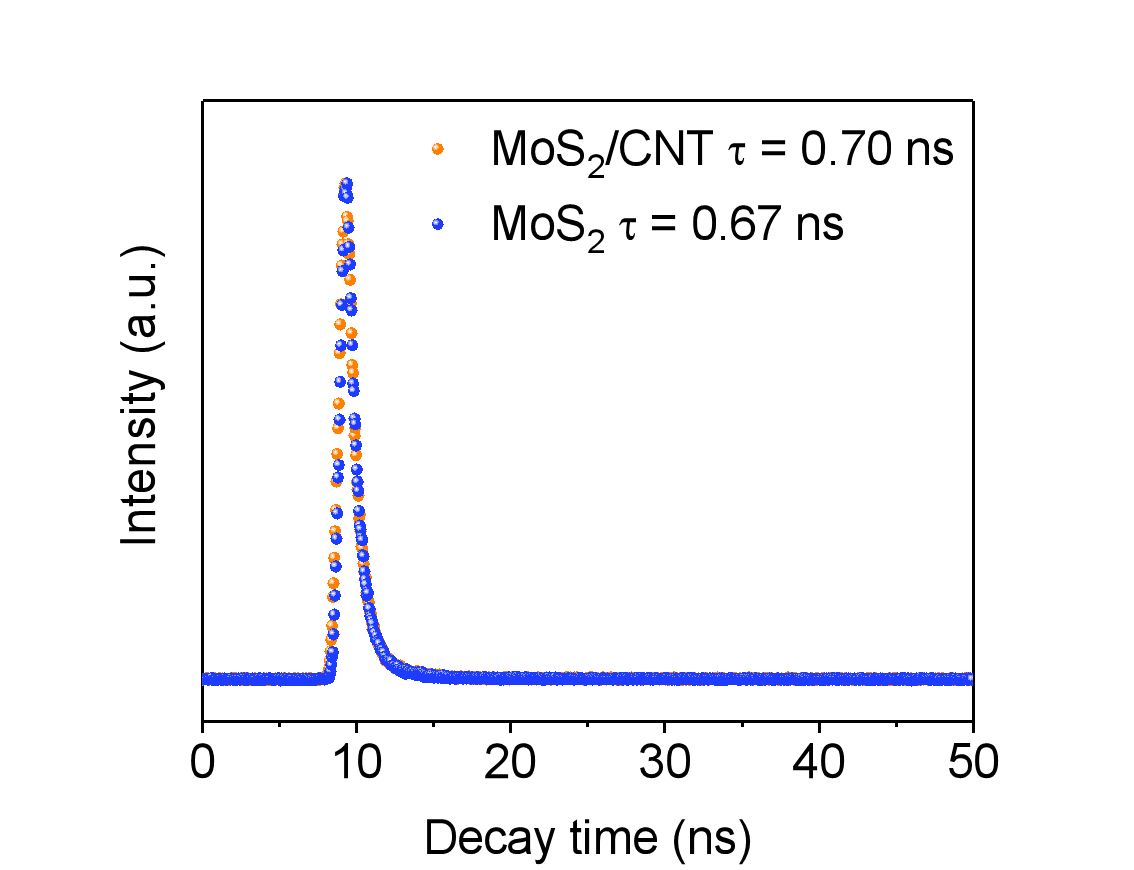


**Fig. S7** Time-resolved transient photoluminescence decay spectra of MoS_2_ and MoS_2_/CNT

First-principles calculations were carried out using density functional theory (DFT) to calculate the kinetic processes under light and no light. According to the intensity value of the Ultraviolet curing lamp in the experiment, the applied electric field component for computation could be calculated by the following equations. According to the intensity value of the Ultraviolet curing lamp in the experiment, the applied electric field component for computation could be calculated by the following equations.

Z_0_ = μ_0_c (S1)

H_0_ = (2S/Z_0_)^1/2^ (S2)

E_0_ = (2Z_0_S)^1/2^ (S3)

Where Z_0_ is the impedance of free space, μ_0_ is the permeability of vacuum (μ_0_= 4π×10^-7^ H/m), c is the light speed (c = 3×10^8^ m/s), S is the electromagnetic wave intensity (S = 42 mW/cm^2^, the intensity of Ultraviolet lamp), H_0_ is the magnetic field intensity, and E_0_ is the electric field intensity. The incident electric field component was calculated to be 563 V/m. To facilitate the subsequent calculation process, the electric field intensity applied in the computation was 600 V/m.

**Table S1** Reaction path and energy changes during the charging process

| Reaction path(charge) | Reaction equation | No lighting (eV) | Lighting (eV) |
| --- | --- | --- | --- |
| a1 | *Li_2_CO_3_ + C = *LiCO_3_ + C + Li | 4.6250 | 4.3285 |
| a2 | *LiCO_3_ + C = *CO_3_ + C + Li | 2.9887 | 2.8845 |
| a3 | *CO_3_ + C = *CO + CO_2_ | -6.4143 | -6.2816 |
| a4 | *Li_2_CO_3_ + CO = *LiCO_3_ + CO + Li | 4.5535 | 4.3064 |
| a5 | *LiCO_3_ + CO + Li = *LiCO_2_ + Li + CO_2_ | -1.8243 | -1.8366 |
| a6 | *LiCO_2_ + Li + CO_2_ = *Li + Li + 2CO_2_ | -0.8993 | -0.8575 |
| a7 | *Li + Li + 2CO_2_ = * + 2Li + 2CO_2_ | 1.4534 | 1.5423 |

**Table S2** Reaction path and energy changes during the discharge process

| Reaction path(discharge) | Reaction equation | No lighting (eV) | Lighting (eV) |
| --- | --- | --- | --- |
| b1 | * + 2Li + 2CO_2_ = *Li + Li + 2CO_2_ | -1.4534 | -1.5423 |
| b2 | *Li + Li + 2CO_2_ = *LiCO_2_ + Li + CO_2_ | 0.8993 | 0.8575 |
| b3 | *LiCO_2_ + Li + CO_2_ = *LiCO_3_ + CO + Li | 1.8243 | 1.8366 |
| b4 | *LiCO_3_ + CO + Li = *Li_2_CO_3_ + CO | -4.5535 | -4.3064 |
| b5 | *CO + CO_2_ = *CO_3_ + C | 6.4143 | 6.2816 |
| b6 | *CO_3_ + C + Li = *LiCO_3_ + C | -2.9887 | -2.8845 |
| b7 | *LiCO_3_ + C + Li = *Li_2_CO_3_ + C | -4.6250 | -4.3285 |


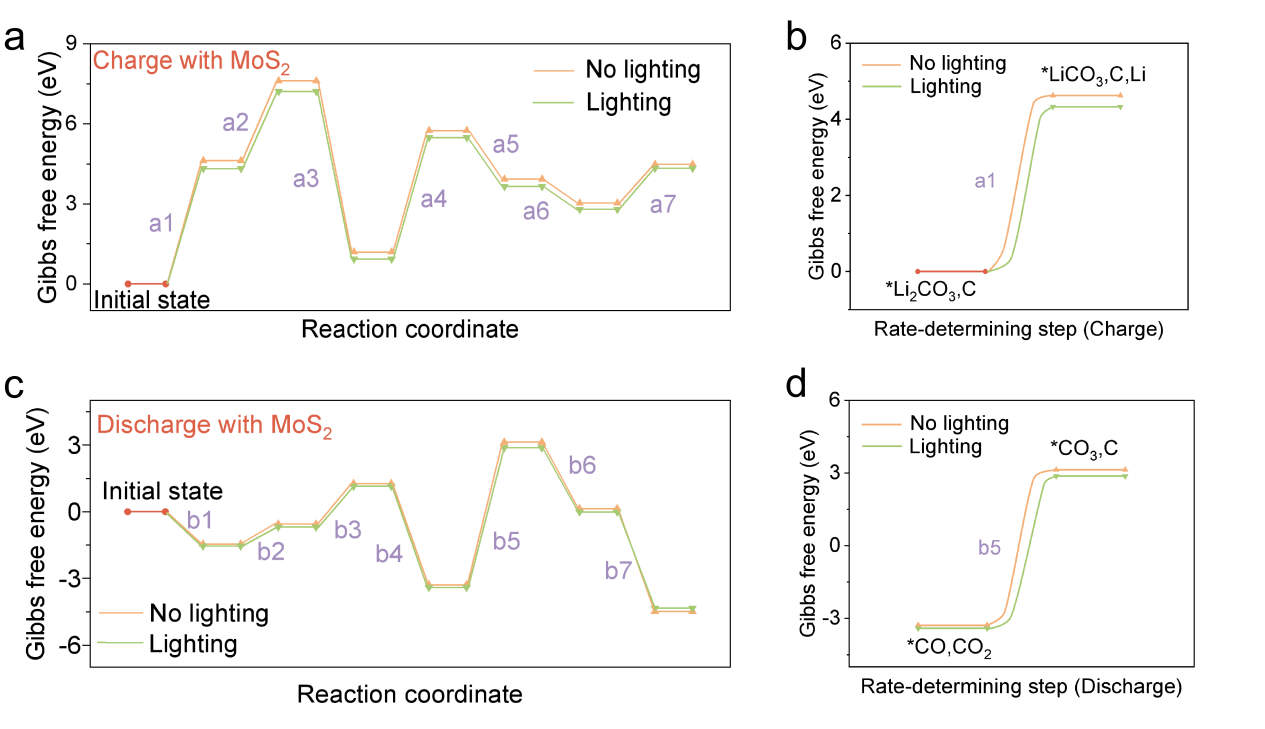


**Fig. S8** (**a**) Charging reaction path with MoS_2_ under lighting and no lighting. (**b**) Rate-determining step in charging reaction. (**c**) Discharge reaction path with MoS_2_ under lighting and no lighting. (**d**) Rate-determining step in discharge reaction


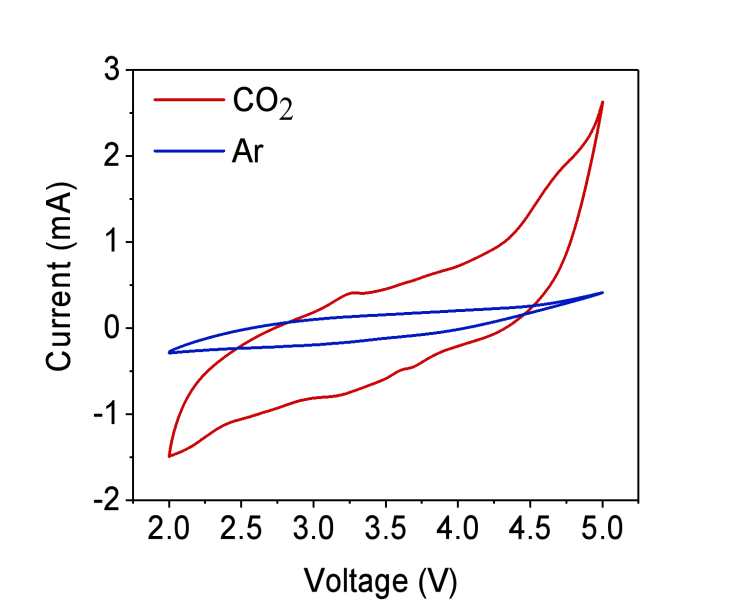


**Fig. S9** Cyclic voltammetry curves of Li–CO_2_ batteries with MoS_2_/CNT cathode in Ar or CO_2_ atmosphere at 1 mV s^–1^


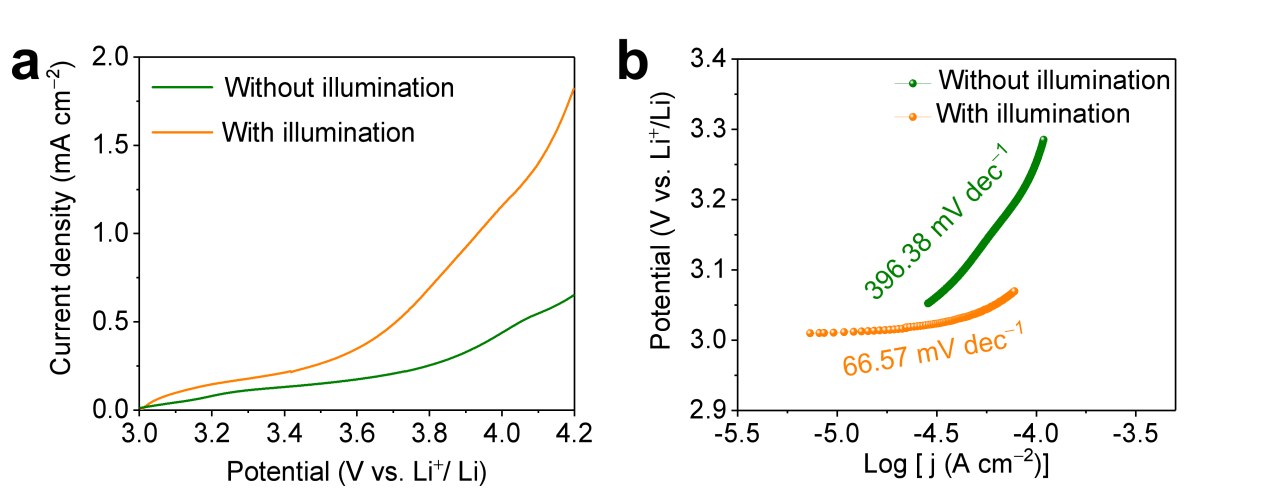


**Fig. S10** (**a**) Linear sweep voltammetry curves in CO_2_ oxidation process at 5 mV s^–1^ and (**b**) corresponding Tafel curves of Li–CO_2_ battery with MoS_2_/CNT cathode in the presence and absence of illumination


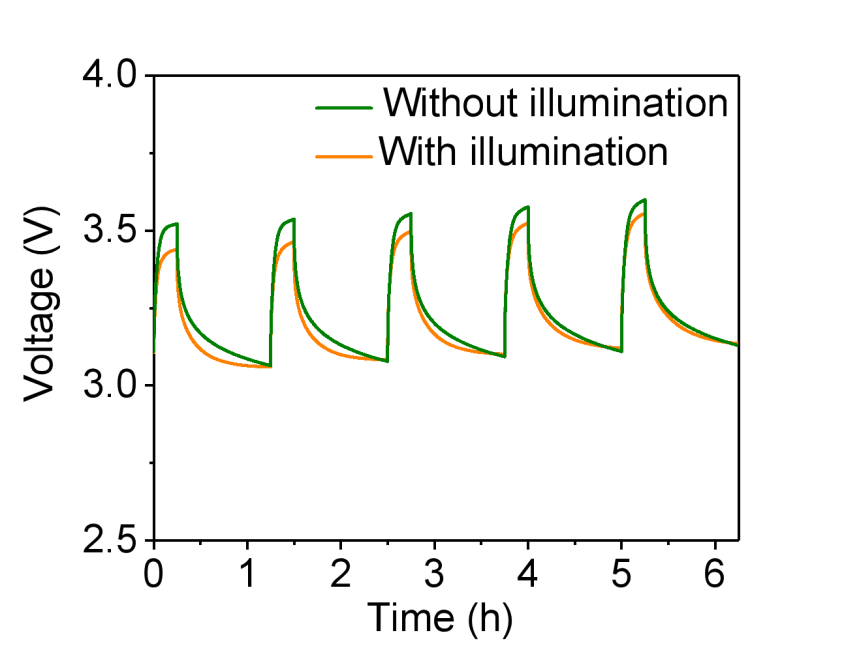


**Fig. S11** Galvanostatic intermittent titration spectra during charge of Li–CO_2_ battery with MoS_2_/CNT cathode with or without illumination


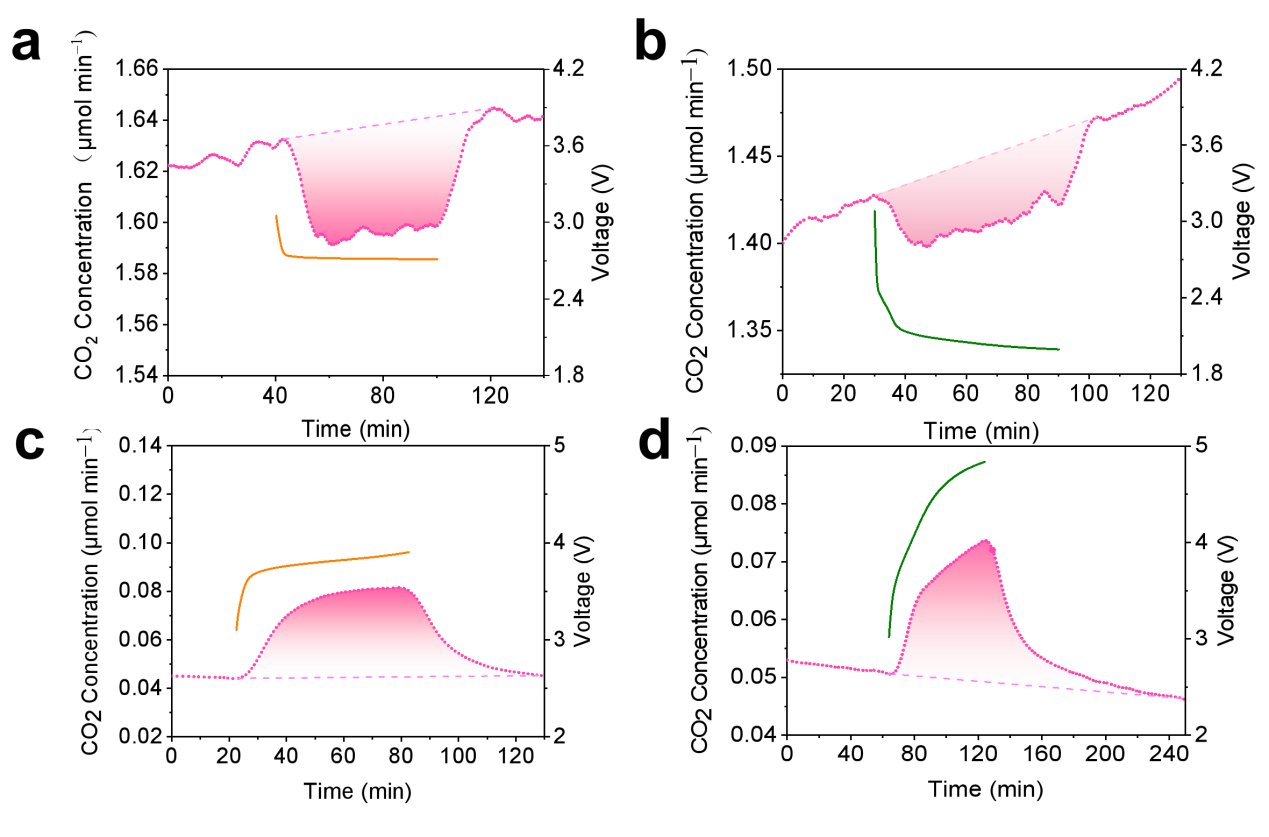


**Fig. S12** Differential electrochemical mass spectrometry results of discharge process (**a**) with and (**b**) without illumination in Li–CO_2_ battery with MoS_2_/CNT cathode. DEMS results of recharge process (**c**) with and (**d**) without illumination in Li–CO_2_ battery with MoS_2_/CNT cathode


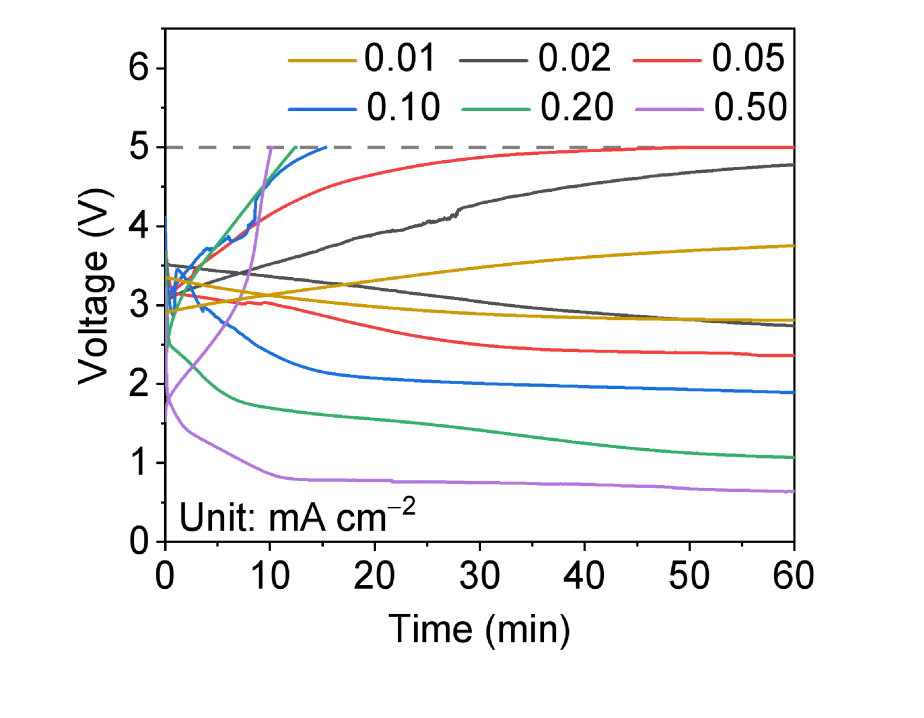


**Fig. S13** Discharge and charge curves of the Li–CO_2_ battery based on the MoS_2_/CNT cathode without illumination at different current density


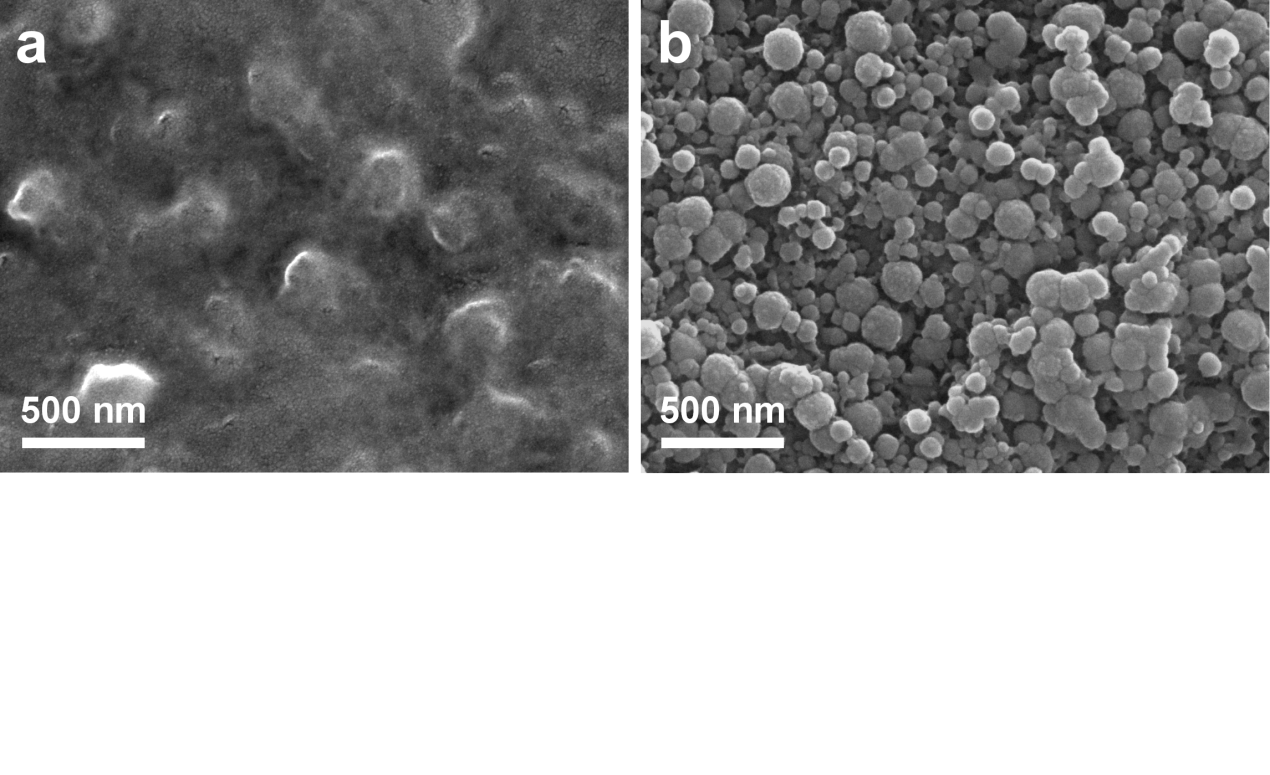


**Fig. S14** Scanning electron microscopy image of MoS_2_/CNT cathode collected from full-discharge Li–CO_2_ battery (**a**) with or (**b**) without illumination


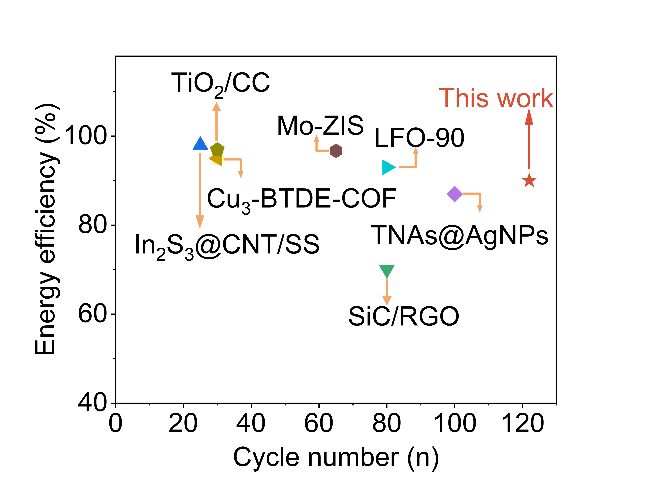


**Fig. S15** Comparison of energy efficiency and cycle performance of Li–O_2_ and Li–CO_2_ batteries with the state-of-the-art cases reported in the literature


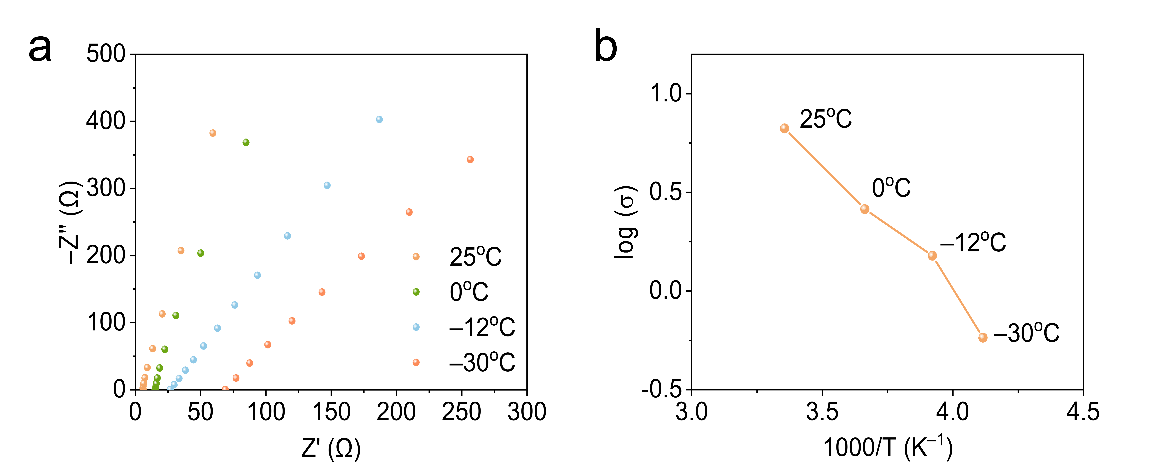


**Fig. S16** (**a**) Nyquist plots and (**b**) ionic conductivity at different temperatures (TEGEDME with 1.0 M LiTFSI is used as electrolyte)


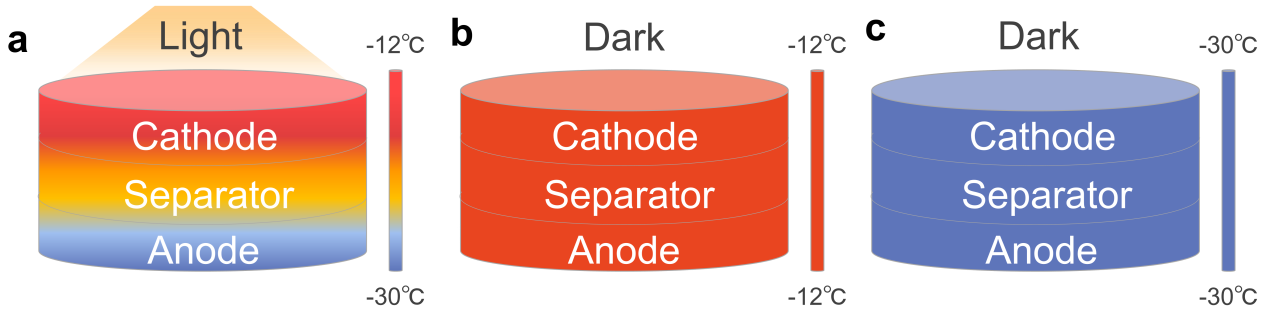


**Fig. S17** Schematic diagram of temperature gradient of Li–CO_2_ battery with MoS_2_/CNT cathode with illumination at –30 ^o^C, without illumination at –30 ^o^C, and without illumination at –12 ^o^C


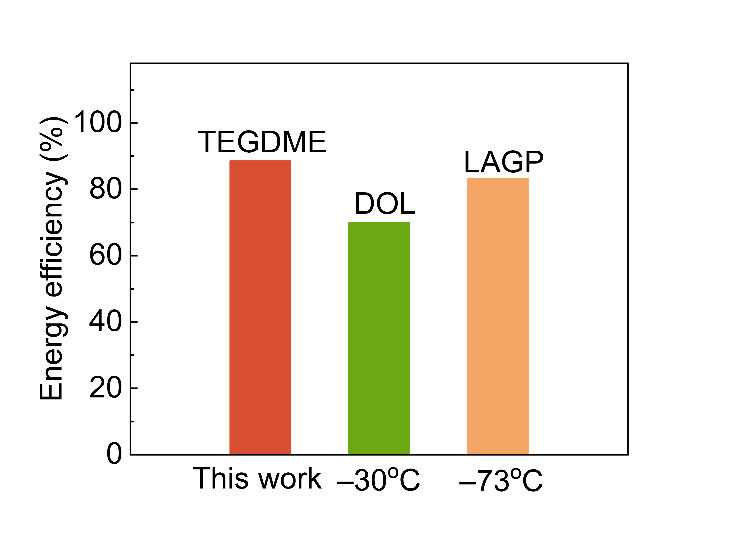


**Fig. S18** Comparison of energy efficiency with different electrolytes (conventional electrolytes, cryo-electrolytes and solid electrolytes) at low temperature


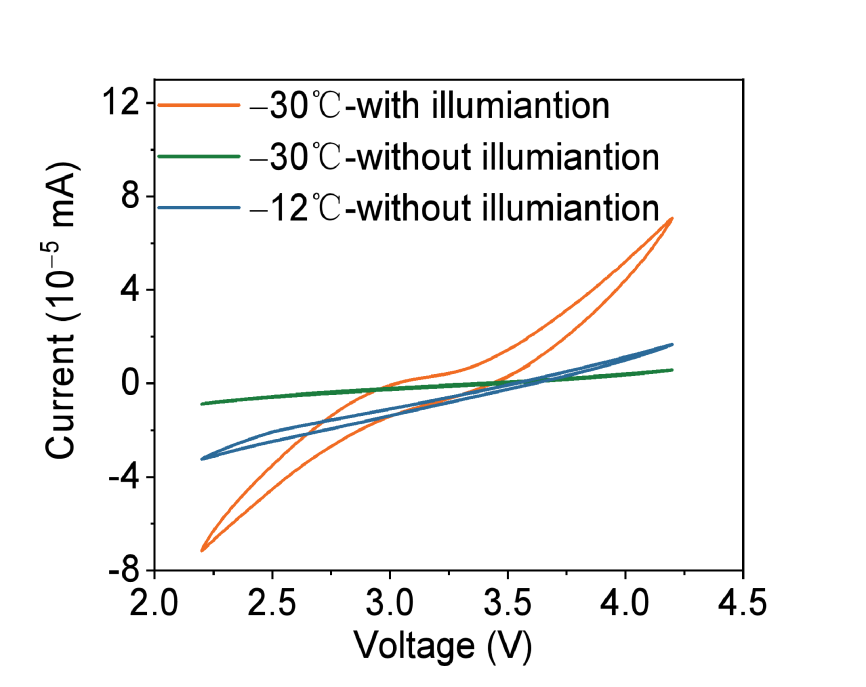


**Fig. S19** Cyclic voltammetry curves of Li–CO_2_ battery with MoS_2_/CNT cathode with illumination at –30 ^o^C, without illumination at –30 ^o^C, and without illumination at –12 ^o^C


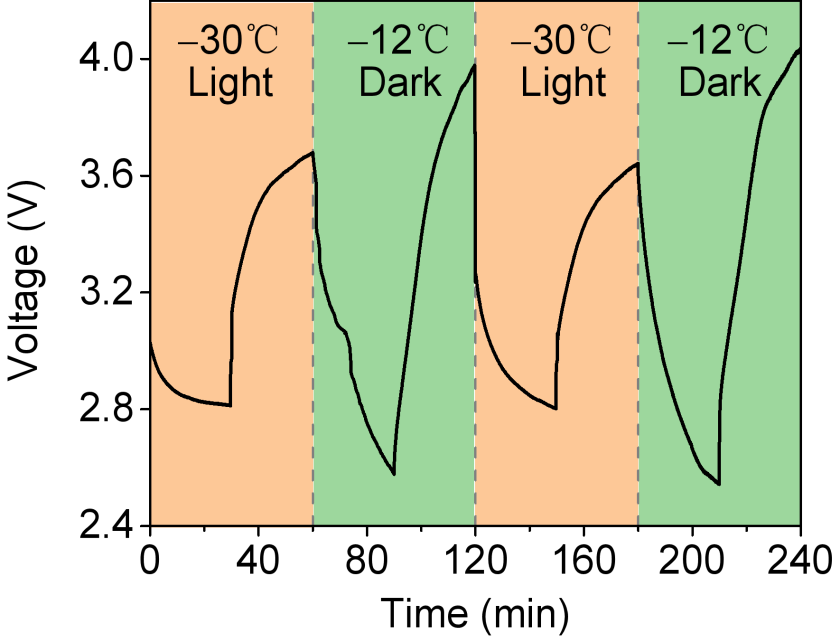


**Fig. S20** Discharge/charge behaviors responding to the Li–CO_2_ battery with illumination at –30 ^o^C and without illumination at –12 ^o^C


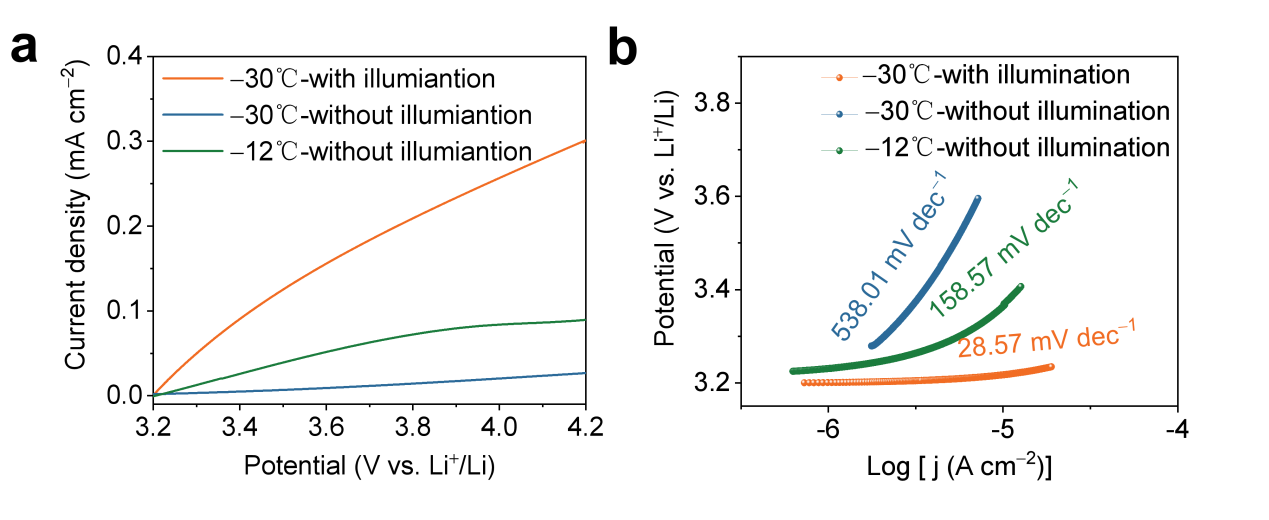


**Fig. S21** (**a**) Linear sweep voltammetry curves in CO_2_ oxidation process and (**b**) Corresponding Tafel curves of Li–CO_2_ battery with MoS_2_/CNT cathode with illumination at –30 ^o^C, without illumination at –30 ^o^C, and without illumination at –12 ^o^C


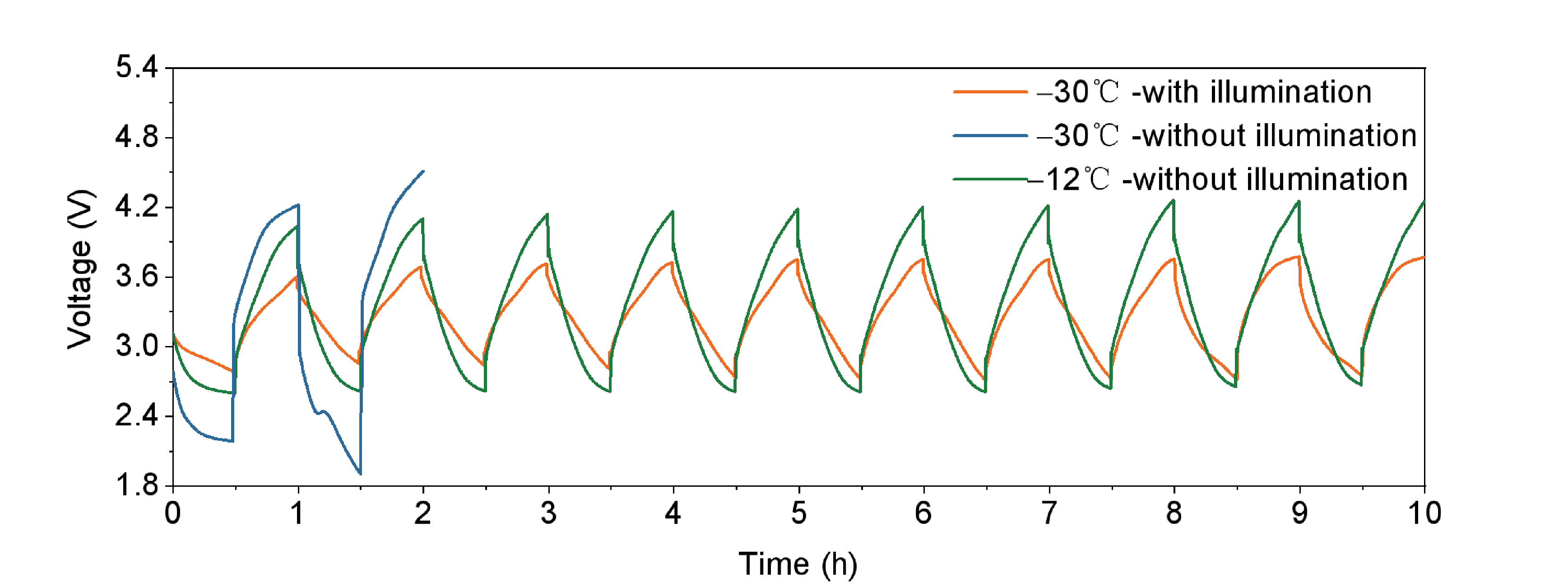


**Fig. S22** Cycling profiles at 0.01 mA cm^–1^ of Li–CO_2_ battery with MoS_2_/CNT cathode with illumination at –30 ^o^C, without illumination at –30 ^o^C, and without illumination at –12 ^o^C


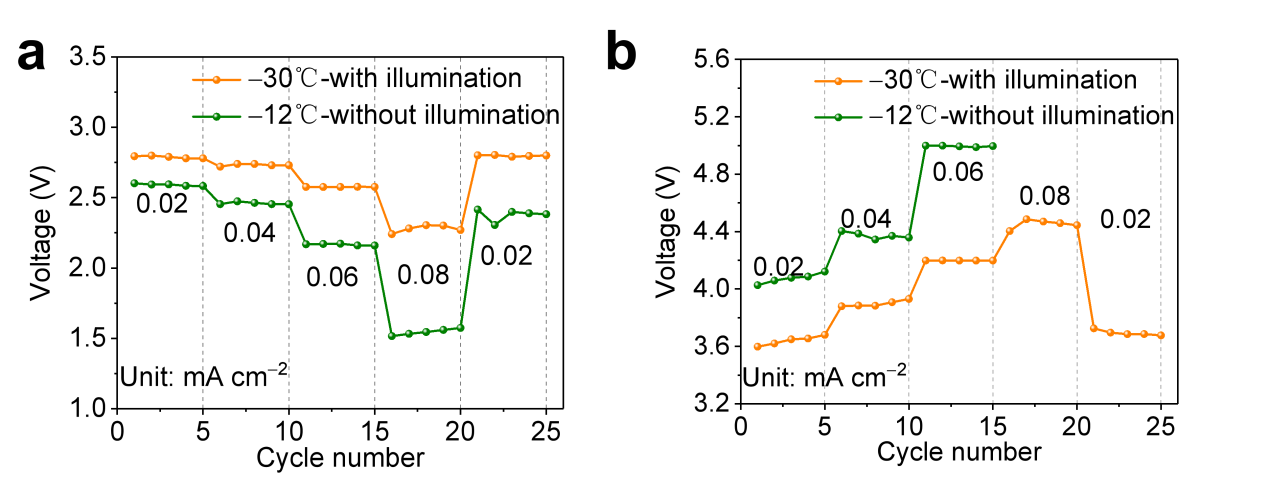


**Fig. S23** (**a**) Discharge rate capability and (**b**) Charge rate capability of Li–CO_2_ battery with MoS_2_/CNT cathode with illumination at –30 ^o^C and without illumination at –12 ^o^C
